# Senescence-associated and immune-related 9p21.3 locus genes in colorectal cancer: epigenetic architecture, molecular landscape and therapeutic possibilities

**DOI:** 10.3389/fcell.2026.1741704

**Published:** 2026-02-11

**Authors:** Darya A. Lisitsa, Vadim V. Shindyapin, Artem R. Nurislamov, Oleg N. Demidov, Daria A. Bogdanova

**Affiliations:** 1 Sirius University of Science and Technology, Sirius, Russia; 2 Institute of Cytology and Genetics, Novosibirsk, Russia; 3 Institute of Cytology RAS, St. Petersburg, Russia; 4 INSERM UMR1231, University of Burgundy, Dijon, France; 5 Bashkir State Medical University, Ufa, Russia

**Keywords:** 9p21.3 locus, *CDKN2A*, *CDKN2B*, colorectal cancer (CRC), senescence, *type I IFNs*

## Abstract

Colorectal cancer (CRC) progression is influenced by genetic and epigenetic aberrations. Oncogenesis of CRC involves the accumulation of mutations in proteins involved in the regulation of cell proliferation, growth and death (Graphical abstract A). DNA methylation has been demonstrated to contribute to tumor initiation, progression, and modulation of therapeutic responses. In this particular landscape, the 9p21.3 locus has been observed to integrate various cellular processes, including cell cycle control (*CDKN2A/CDKN2B* and ANRIL), immune signaling (cluster of *type I interferons*), and metabolic regulation (*MTAP, MLLT3*). This creates relationships that may affect tumor intrinsic and extrinsic features, immunogenicity, and therapeutic sensitivity. The objective of our analysis is to provide a comprehensive overview of the role of the 9p21.3 locus in CRC, focusing on its potential implications for treatment decisions and prediction of treatment responses. Analyzing the 9p21.3 status would help stratify CRC patients into different groups and guide the choice of personalized therapy for CRC. It could also enhance current CRC treatment by pretreating patients with demethylating agents and using an immunotherapeutic approach in combination with senolytic drugs (Graphical abstract B).

## Graphical Abstract

**Figure:**
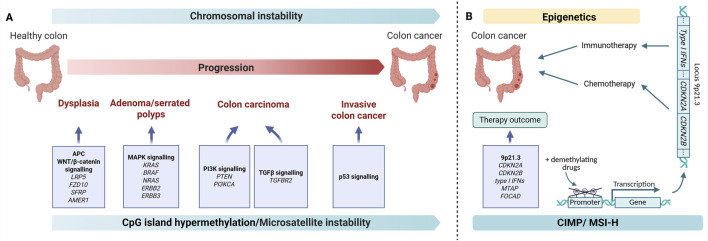
Graphical abstract. **(A)** Stages of CRC oncogenesis. The gradual accumulation of mutations in intestinal cells leads to disruptions in signaling pathways (WNT, MAPK, PI3K, TGFb, p53) and leads to malignancy **(B)** Epigenetic changes in the 9p21.3 locus can impact the outcome of CRC therapy. Demethylating may restore gene expression at the 9p21.3 locus and enhance the effects of chemotherapy and immunotherapy. Illustration created with BioRender.com.

## 1 Introduction

Colorectal cancer (CRC) remains a major global health burden, ranking third in incidence and second in mortality, with >1.9 million new cases and ∼904,000 deaths estimated in 2022 (Bray et al., 2024). Beyond classical genetic drivers, CRC is profoundly shaped by epigenetic dysregulation, particularly DNA methylation, which contributes to tumor initiation, progression, and therapeutic response (Hinoue et al., 2012; Rawson and Bapat, 2012; Vedeld et al., 2018). The CpG island methylator phenotype (CIMP) delineates a distinct molecular class characterized by widespread promoter hypermethylation; CIMP-high tumors overlap with MSI-H disease. MSI-H refers to tumors with instability in ≥30% of tested microsatellites. These categories are not fully congruent, underscoring the need for integrated molecular stratification (Weisenberger et al., 2006; Hinoue et al., 2012).

CIMP exhibits significant molecular heterogeneity beyond the traditional binary classification (Weisenberger et al., 2006). Large-scale studies have identified CIMP-high (CIMP-H) and CIMP-low subtypes with distinct biological behaviors. CIMP-H tumors (≥3 of 5 methylated markers in the Weisenberger panel) show concordant methylation patterns across tumor regions with >95% intratumoral consistency, indicating clonal epigenetic alterations. However, individual marker variability occurs in approximately one-third of CIMP-positive cases, suggesting complex methylation dynamics (Nosho et al., 2008; Flatin et al., 2021).

There are well-established epigenetic biomarkers of colorectal cancer, like *MLH1* promoter hypermethylation that leads to mismatch repair (MMR) deficiency and MSI (Nguyen et al., 2020; Joo et al., 2023), *SEPT9* methylation that leads to enhanced cell proliferation and migration, *etc.* In our review we decided to focus on the 9p21.3 locus (Leerhoff et al., 2023; Cao et al., 2024).

The 9p21.3 locus is of special interest. This ∼0.5-Mb region encompasses *CDKN2A/CDKN2B* (encoding p16^INK4a^/p14^ARF^ and p15^INK4b^), the lncRNA ANRIL, and *MTAP*, and lies adjacent to a dense *type I interferon* gene cluster–elements with direct relevance to cell-cycle control, cellular senescence, tumor immunogenicity, and response to therapy (Hinoue et al., 2012; Barriga et al., 2022; Morgan et al., 2023). Epigenetic silencing of *CDKN2A/CDKN2B* gene cluster constrains antitumor checkpoints, whereas deletions at 9p21.3 can remove interferon genes, promoting an immune-cold microenvironment and resistance to immune-checkpoint blockade (Morgan et al., 2023). Collectively, findings from loss-of-function deletion studies of 9p21.3 support a therapeutic strategy in which DNA-demethylating agents are used to re-activate epigenetically silenced tumor-suppressor and type I interferon pathways–provided the locus remains structurally intact without large-scale genomic deletions that would eliminate the DNA methyltransferase inhibitor target sequences (Chiappinelli et al., 2015; Roulois et al., 2015; Topper et al., 2020; Barriga et al., 2022).

Previous studies have focused on the role of 9p21.3 aberrations in other cancer types. Here, we describe recent evidence of the important role of 9p21.3 epigenetic changes in colorectal cancer (CRC). CRC is also characterized by the methylation of regions of the genome other than 9p21.3 that play an important role in CRC pathogenesis and are responsible for specific CRC subtypes, such as CIMP. The genes affected by CIMP and their roles in CRC are described in detail in other papers, and we briefly mention them in the current work, which is devoted to the role of 9p21.3 in CRC. The status of locus 9p21.3 may affect the expression of genes associated with intestinal tumor formation. These genes play an important role in determining tumor cell characteristics, shaping the tumor microenvironment, and influencing the antitumor immune response. Overall, changes in 9p21.3 methylation may have high prognostic value and influence patient stratification alongside other markers in the future. Based on accumulated data, we propose expanding possible therapeutic strategies. Demethylation therapy can restore gene expression at the 9p21.3 locus, potentially enhancing the efficacy of chemotherapeutic and immunotherapeutic drugs in certain patient cohorts (Mansfield et al., 2024; Alum et al., 2025; Kim, 2025; López et al., 2025; Suraweera et al., 2025). The integration of senolytic agents into treatment regimens is a hypothetical concept primarily based on preclinical data and requires thorough clinical validation with appropriate biomarker stratification before therapeutic implementation. This review summarizes the current evidence on the epigenetics of 9p21.3 in CRC and its impact on treatment outcomes. We also justify clinical strategies that personalize treatment approaches for patients based on genetic and epigenetic data.

## 2 The role of 9p21.3 genes in the pathogenesis of colorectal cancer

**FIGURE 1:**
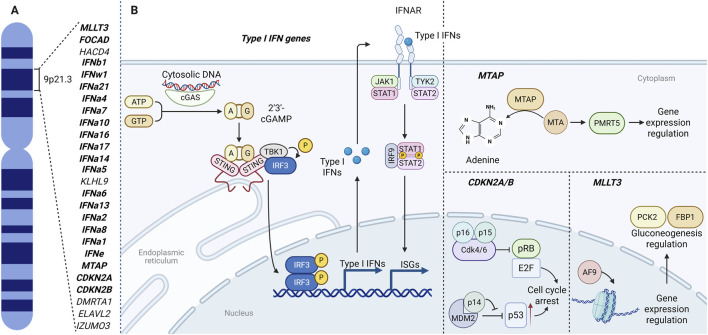
The 9p21.3 locus: gene map **(A)** and integrated functional circuits **(B)** linking cGAS–STING/type I IFN signaling, IFNAR–JAK–STAT transcription, CDKN2A/B (p16^INK4a^, p14^ARF^, p15^INK4b^) cell-cycle control, MTAP–PRMT5 methylation, and MLLT3(AF9)-mediated chromatin–metabolic regulation through PCK2 and FBP1. Illustration created with BioRender.com. Parts of the layout were adapted from BioRender templates (Iwasaki, 2025; Lugano, 2025).

The association between the short arm of chromosome 9 (9p) and cancer was recognized more than 3 decades ago (Hayashi et al., 1991). More recent work has refined this link and focused attention on the 9p21.3 interval in particular (Han et al., 2021; Barriga et al., 2022). This ∼0.5 Mb region contains a dense cluster of *type I interferon (type I IFN)* genes (*IFN-I* gene cluster), several tumor-suppressor genes–including *CDKN2A, CDKN2B, CDKN2B-AS1* (ANRIL), *MTAP*, *MLLT3, FOCAD*–and other genes with less well-defined roles in oncogenesis, such as *ELAVL2*, *HACD4*, *KLHL9*, *DMRTA1*, *IZUMO3* (Figure 1A) (Spiliopoulou et al., 2022).

Because homozygous deletions at 9p21.3 rank among the most frequent somatic copy-number alterations across human cancers (Cox et al., 2005; Li W.-Q. et al., 2014), much of the historical emphasis has been on loss-of-function *via* deletion. In CRC, however, 9p21.3 dysfunction is not limited to structural loss: epigenetic silencing can downregulate locus genes and partially phenocopy deletion with distinct implications for prognosis and treatment.

Systematically defining the roles of 9p21.3 genes in CRC pathogenesis will enable stratification by locus epigenetic status and prescribe the use of demethylating strategies to reactivate gene expression. In the following chapters we will focus primarily on the gene-by-gene functions of 9p21.3 in CRC and other malignancies to highlight additional mechanisms that could affect CRC.

### 2.1 *CDKN2A* and *CDKN2B* genes

The *CDKN2A* gene encodes two proteins: p16^INK4a^ and an alternative reading frame gene product p14^ARF^ (Cilluffo et al., 2020). These proteins are tumor suppressors that regulate the cell cycle. The *CDKN2B* gene is another gene that encodes tumor suppressor–p15^INK4b^ (Gil and Peters, 2006). *CDKN2A* and *CDKN2B* genes are located in the INK4/ARF locus (Kim and Sharpless, 2006; Farooq and Notani, 2022; 2022).

p16^INK4a^ and p15^INK4b^ are inhibitors of cyclin-dependent kinase CDK4/6. Both p16^INK4a^ and p15^INK4b^ prevent the phosphorylation of the retinoblastoma protein (RB). The stable non-phosphorylated form of RB binds to the transcription factor E2F, to suppress the expression of cell-cycle-related genes. E2F inactivation leads to an arrest in the cell’s transition from G1 phase to S phase (Figure 1B) (Zhao et al., 2016).

p14^ARF^ is one of the proteins that regulates another tumor suppressor–p53. Normally, p53 is rapidly ubiquitinated by MDM2 and degraded by proteasomes, but when MDM2 binds to p14^ARF^, p53 is no longer degraded. This can lead both to cell cycle arrest and apoptosis (Figure 1B) (Ozenne et al., 2010; Zhou et al., 2023).

p16^INK4a^, p14^ARF^ and p15^INK4b^ can stop the cell cycle in response to stress insults, giving cell time for restoration. Also, p16^INK4a^ has been reported to enhance cell viability and migration in CRC by inhibiting cuproptosis, a type of cell death caused by excess copper accumulation (Cheng et al., 2024).

Deletion and promoter hypermethylation of *CDKN2A* and *CDKN2B* are common in various cancers, in CRC in particular (Zhao et al., 2016). *CDKN2A* hypermethylation is the most studied epigenetic marker in the context of CRC compared to other genes of the 9p21.3 locus. It is reported that *CDKN2A* promoter hypermethylation in CRC is observed in 30% of CRC patients, and loss of expression in 25% of all cases (Shima et al., 2011). The methylation rate of *CDKN2A* is comparable to methylation rate of recognized markers such as *MLH1*, the gene encoding the DNA mismatch repair protein. *MLH1* is methylated in 20%–25% of colorectal cancer cases (Moreno-Ortiz et al., 2021; Rico-Méndez et al., 2025). Despite the high specificity of *CDKN2A* methylation as a marker of CRC (96%–100%) (Fatemi et al., 2022), its sensitivity is lower than that of other diagnostic markers of CRC, such as *SEPT9* or *SFRP2* (Fatemi et al., 2022; Oh and Cho, 2024). That makes *CDKN2A* methylation more suitable for confirming diagnosis rather than primary screening. *CDKN2A* promoter hypermethylation in CRC patients has been shown to be associated with invasion, metastasis, and overall worse prognosis (Xing et al., 2013).

In CRC, *CDKN2B* is frequently inactivated by promoter hypermethylation (e.g., 26% in a Japanese cohort with concordant mRNA reduction and adverse features) and, less commonly, by 9p21.3 deletions. Reported methylation frequencies vary widely across populations and can be as high as 68% in some Chinese cohorts (Xu et al., 2004; Ishiguro et al., 2006; Nieminen et al., 2012).

Dysfunction of *CDKN2A/B* can lead to uncontrolled tumor cell growth and poor prognosis. Li Yang et al. showed that hypermethylation of the *CDKN2A* promoter in a mouse model of CRC leads to remodeling of the tumor microenvironment through increased PD-L1 expression and poorer prognosis (Yang et al., 2023). Overall, mutations in *CDKN2A* may contribute to poor prognosis in a variety of cancer types (Debniak et al., 2005; Yang et al., 2016; Horn et al., 2021; Li C. et al., 2022).

When *CDKN2A* dysfunction in CRC is caused by hypermethylation, restoration of normal expression by demethylating agents may be used as anti-cancer drugs. Moreover, they may be more effective when used in combination with anti-PD-L1 immunotherapy, as immunotherapy is less effective in the presence of *CDKN2A* epimutation (Yang et al., 2023).

Although low expression or loss of *CDKN2A/B* is a marker of poor prognosis in CRC, overexpression also has negative consequences. It has been shown that *CDKN2A* can promote invasion and metastasis through the regulation of E-cadherin, N-cadherin and vimentin expression (Shi et al., 2022). Thus, aberrant *CDKN2A/B* expression, whether consistently reduced or enhanced, can lead to adverse consequences. On the other hand, it has been demonstrated that high *CDKN2A* expression correlates with high response to immunotherapy (Dong et al., 2023).

In addition to the classical functions of cyclin-dependent kinase inhibitors in tumors described above, they can influence the formation of certain structures in the tumor. Jie Fan et al. demonstrated the role of *CDKN2A* in the formation of “neutrophil-in-tumor” (hNiT) structures in oropharyngeal squamous cell carcinoma. The formation of hNiT is associated with an unfavorable prognosis. It was shown that p16^INK4a^ expression in HPV-associated oropharyngeal squamous cell carcinoma correlated with decreased hNiT formation and a more favorable prognosis (Fan et al., 2022).

A transient, therapeutically induced increase in cell-cycle inhibitor expression may confer clinical benefits. In contrast, sustained overexpression can be harmful. Therefore, treatment should be time-limited and targeted only at *CDKN2A/B*-negative tumor cells.

### 2.2 *Type I IFN* genes

Next to *CDKN2A* and *CDKN2B* genes, *type I IFN* gene cluster is located at the locus 9p21.3. It includes 13 subtypes of *IFNα*, *IFNβ1*, *IFNε* and *IFNω1* genes (UCSC Genome Browser, 2025).

Type I IFNs are mostly in charge of response against viral infections. When a cell encounters a virus, viral nucleic acids are recognized with a pattern recognition receptor (PRR). The recognition triggers various signaling cascades that lead to the synthesis of type I IFNs (Swiecki and Colonna, 2011; McNab et al., 2015; Mödl et al., 2023).

Type I IFNs bind to the IFNAR receptor, leading to the activation of the JAK1 and TYK2 kinases. These kinases phosphorylate the transcription factors STAT1 and STAT2 which dimerize and translocate from the cytoplasm to the nucleus. There, they bind to IRF9 to form a complex that triggers the expression of ISGs–interferon-stimulated genes (Figure 1B). Signal transmission can also be carried out through other STATs (Ivashkiv and Donlin, 2014; McNab et al., 2015; Mödl et al., 2023).

The key signaling pathway associated with the expression of type I IFNs is the cGAS-STING pathway. This mechanism is responsible for the recognition of double-stranded viral and bacterial DNA and, most importantly in the context of this review, DNA of damaged cells in the cytoplasm. The initiator enzyme is cyclic GMP-AMP synthase (hereinafter cGAS). It detects foreign and unnaturally located DNA in the cytoplasm, dimerizes and triggers the formation of cGAMP. cGAMP binds to STING, an adapter protein located on the ER. Interaction with cGAMP causes conformational changes in STING and leads to the formation of a STING complex with TBK1, NIK and IKK kinases. This complex triggers a number of phosphorylation reactions and, among other things, leads to the expression of type I IFNs, particularly IFNβ (Figure 1B) (Corrales et al., 2016; Galluzzi et al., 2018; Chin et al., 2023; Luo et al., 2024). Moreover, tumor cells can secrete cGAMP externally, triggering an interferon response in cells of the tumor microenvironment (Samson and Ablasser, 2022). It is also assumed that DNA from destroyed tumor cells can trigger the cGAS-STING pathway in immune cells, particularly phagocytes (Samson and Ablasser, 2022).

The role of the cGAS-STING pathway and type I IFNs in tumors is controversial. On one hand, due to the increased production of type I IFNs by tumor cells, immune cells are activated: dendritic cells, NK cells, T cells (Mender et al., 2020; Samson and Ablasser, 2022). Additionally, type I IFNs inhibit MDSCs (Mödl et al., 2023). This provides a comprehensive antitumor response. Type I IFNs can also induce apoptosis in cancer cells, including CRC (Zhou et al., 2020; Musella et al., 2021; Mödl et al., 2023). It is reported that type I IFNs can contribute to cell cycle arrest through induction of cyclin-dependent kinase inhibitors p15^INK4b^, p21^Cip1^ and p27^Kip1^ (Musella et al., 2021; Mödl et al., 2023). Type I IFNs can also act as negative regulators of VEGF signaling, preventing angiogenesis. In particular, it was shown that IFNα can lower vascularization level of the CRC liver metastases (Mödl et al., 2023). However, it has been reported that at later stages of tumor development, type I IFNs may, on the contrary, have a pro-tumor effect by increasing the production of immunosuppressive molecules such as PD-L1, IDO, and IL-10 (Zhou et al., 2020). In CRC, decreased IFNAR expression and impaired interferon signaling may be observed, which may lead to altered cancer-associated fibroblast (CAF) function and tumor matrix remodeling (Sun et al., 2025). Type I IFNs play a crucial role in tumor control by promoting dendritic cell maturation and enhancing antigen cross-priming to activate T cells (Su et al., 2019). Cancer cell intrinsic type I IFNs signaling modulates therapeutic responses, influencing outcomes to chemotherapy (Sistigu et al., 2014), radiotherapy (Deng et al., 2014), and immunotherapy (Su et al., 2019).

### 2.3 *MTAP* gene

*MTAP* encodes methylthioadenosine phosphorylase, a crucial enzyme in purine and methionine metabolism. MTAP converts MTA (5′-methylthioadenosine), which is formed during methionine metabolism, into adenine, thereby regulating its intracellular levels. If MTAP is not functioning, MTA accumulates. It was demonstrated in glioblastoma cell line models that the accumulation of MTA may result in suppression of T-cell activity, decreased response to interferons and instructing the tumor microenvironment and macrophage polarization toward M2 (Han et al., 2021). MTA inhibits the PRMT5 (protein arginine methyltransferase 5), a methyltransferase responsible for epigenetic regulation on the histone methylation level, methylation of transcription factors, *etc.* (Figure 1B) (Patro et al., 2022; Bedard et al., 2023; Ngoi et al., 2024).

In fact, *MTAP* deletion or inactivation is a vulnerability for tumor cells because MTA accumulation combined with using PRMT5 inhibitors will lead to selective destruction of cancer cells without MTAP. However, there are several problems with using the described mechanism in therapy. Firstly, it is reported that cancer cells (e.g., glioblastoma) can lower the level of MTA by eliminating it from the cell (Barekatain et al., 2021). The second issue is the rare occurrence of MTAP loss in CRC. MTAP loss is widespread in gastrointestinal cancers, but it is more common for upper gastrointestinal cancers. *MTAP* is often deleted together with *CDKN2A/B* due to their neighboring localization within locus 9p21.3. (Bedard et al., 2023; Mauri et al., 2024). Despite the rarity of deletion in CRC, *MTAP* could be found mutated in this type of cancer. It is assumed that the loss of *MTAP* does not have a significant impact on the patient’s prognosis (Mauri et al., 2024).

### 2.4 *MLLT3* gene

The *MLLT3* gene encodes protein AF9 sharing the YEATS domain with other proteins of the YEATS family. YEATS domain can bind to acetylated and crotonylated lysines, due to which AF9 acts as a chromatin reader and is involved in the regulation of transcription (Li Y. et al., 2014; 2016; He et al., 2023).

Xuefeng He et al. (He et al., 2023) showed that AF9 plays an important role in the epigenetic regulation of genes coding gluconeogenesis enzymes PCK2 and FBP1 (Figure 1B). PCK2 is a mitochondrial isoform of PEPCK–phosphoenolpyruvate carboxykinase. It can help in tumor progression providing metabolic plasticity in conditions of glucose deficiency (Grasmann et al., 2019). On the other hand, FBP1 (fructose 1,6-bisphosphatase 1) acts as a tumor suppressor. It is reported that FBP1 can inhibit glycolysis (Grasmann et al., 2019).

The predominant metabolic process in CRC is glycolysis (Graziano et al., 2017; Lu et al., 2021), while gluconeogenesis is found to be less active (Grasmann et al., 2019; Wang and Dong, 2019). According to Xuefeng He et al., a decrease in AF9 expression led to a decrease in PCK2 and FBP1 expression, and consequently, a decrease in gluconeogenesis. Thus, metabolic reprogramming in CRC may be associated with AF9 expression and activity. Currently there is no clear data demonstrating common mutations or epimutations associated with the *MLLT3* gene in CRC. It is shown that as CRC progresses, AF9 expression decreases, with the risk of relapse increasing in patients with lower AF9 levels (He et al., 2023).

### 2.5 *FOCAD* gene

Another gene of the 9p21.3 locus, *FOCAD*, encodes the focal adhesion protein (focadhesin), which is involved in cell adhesion and regulation of the cell cycle, and also acts as a tumor suppressor (Brand et al., 2020; Harmonizome 3.0: FOCAD, 2025). The role of focadhesin in tumors was demonstrated using gliomas as a model. Focadhesin can bind to tubulin and reduce the rate of microtubule assembly, which reduces tumor cell migration (Brand et al., 2020). Moreover, focadhesin was shown to accumulate in the G2/M phase and slow it down, which may explain its relationship with cell cycle regulation (Brand et al., 2020).

In a non-small-cell lung cancer model, it was shown that a signaling pathway involving focadhesin can increase the sensitivity of cells to ferroptosis (Liu et al., 2020).

The *FOCAD* gene is expressed in the proliferating epithelial cells of the colonic crypts. Several studies have shown that *FOCAD* deletions are associated with polyposis and the development of CRC (Weren et al., 2015; Belhadj et al., 2020). On the contrary, some point mutations do not initiate oncogenesis, unlike deletions of the gene regions and some missense mutations found in patients with CRC (Belhadj et al., 2020). Further studies are needed to determine the specific role of *FOCAD* in the development of polyposis and CRC.

### 2.6 Clinical context and biomarker applications

The genetic and epigenetic aberrations, as well as the role of genes located on 9p21.3 in tumorigenesis, render this chromosomal region an intriguing therapeutic target in the context of CRC. The clinical significance of this phenomenon is particularly pronounced in MSI-H CRC, where the intersection of DNA methylation patterns with immune phenotypes creates opportunities for highly effective targeted therapeutic intervention.

*CDKN2A/B* hypermethylation demonstrates differential prevalence between molecular subtypes of CRC, with *CDKN2A* promoter hypermethylation detected in approximately 30% of CRC cases overall using validated MethyLight methodology (Shima et al., 2011). This hypermethylation occurs as part of the broader epigenetic dysregulation characteristic of the CIMP, which shows strong association with MSI-H status (Kim et al., 2024).

*CDKN2A* methylation status correlates independently with CIMP classification, with hypermethylated tumors exhibiting significantly increased odds of CIMP-high status (Shima et al., 2011), showing an OR of 39.6 (95% CI, 20.6–76.1) for CIMP-high compared to CIMP-0, and an OR of 5.30 (95% CI, 3.52–8.00) for CIMP-low compared to CIMP-0 (Shima et al., 2011). This relationship establishes 9p21.3 methylation as both a component and potential surrogate marker of epigenetic dysregulation patterns that influence therapeutic responsiveness.

While *CDKN2A* promoter methylation occurs in approximately 30% of CRC cases, direct comparison with established biomarkers reveals both complementary and distinct clinical utilities (Shima et al., 2011; Rico-Méndez et al., 2025). *MLH1* promoter hypermethylation, detected in 20%–25% of sporadic CRCs, represents the predominant mechanism underlying MSI-H phenotype in the absence of germline mutations. A recent systematic analysis of 138 CRC tumors demonstrated that *MLH1* methylation (21% partial, 3.6% complete) showed significant concordance with MSI-H status (p < 0.01) when assessed across five distinct CpG island regions. In contrast, *CDKN2A* methylation exhibits broader distribution across molecular subtypes, occurring in both MSI-H (23%) and microsatellite stable/low (MSS/MSI-L, 13.4%) tumors, positioning it as a CIMP-associated rather than MSI-specific marker (Kim et al., 2024). The prevalence of *CDKN2A* promoter methylation in CRC is approximately 30%, and as a standalone marker its diagnostic utility remains lower than that of blood-based methylated SEPT9 assays (pooled sensitivity ≈69–71% and specificity ≈91–92% in meta-analyses) or combined multi-gene methylation panels, while *CDKN2A* methylation shows high specificity around 98% (Shima et al., 2011; Nian et al., 2017; Karam et al., 2019; Zhao et al., 2019; Hariharan and Jenkins, 2020). Notably, *MLH1* promoter methylation has dual diagnostic and therapeutically relevant roles: it supports identification of sporadic MSI-H colorectal cancers, a subgroup that is generally responsive to PD-1–based immune checkpoint inhibition, while also distinguishing these cases from Lynch syndrome for whom germline testing is indicated within standardized diagnostic pathways (McRonald et al., 2024; Eslinger et al., 2025; Rico-Méndez et al., 2025). Recent population-level data from England show that only 44% of colorectal cancers undergo dMMR screening, with approximately four-fold geographic variation, underscoring implementation gaps even for this established biomarker. These observations support using *CDKN2A* methylation analysis as complementary–rather than a replacement–to MLH1 testing, particularly for stratifying CIMP-high subsets in which *CDKN2A* and *MLH1* promoter methylation are components of validated CIMP panels and are associated with distinct immune phenotypes (e.g., higher CD8^+^ TIL densities and PD-L1 expression in CIMP-high MSI-H tumors) (Ogino et al., 2007; Kim et al., 2024).

The clinical context for 9p21.3-targeted interventions builds upon well-established immunotherapy efficacy in MSI-H CRC. Recent Phase III data demonstrate superior efficacy of immune checkpoint inhibition in this molecular subtype. The KEYNOTE-177 trial established pembrolizumab as first-line standard of care, showing superior progression-free survival compared to chemotherapy (median 16.5 *versus* 8.2 months; HR 0.60; 95% CI 0.45–0.80; P = 0.0004) (Casak et al., 2021), leading to FDA approval on 29 June 2020. The CheckMate 8HW trial provided definitive evidence for nivolumab plus ipilimumab as an alternative first-line option (Andre et al., 2024), demonstrating 79% reduction in progression risk compared to chemotherapy (HR 0.21; 97.91% CI 0.13–0.35; P < 0.0001) (Lenz et al., 2024). Recent comprehensive meta-analyses have confirmed the superior efficacy of combination immunotherapy, with nivolumab plus ipilimumab demonstrating significantly improved progression-free survival in MSI-H CRC patients (HR 0.676; 95% CI: 0.583–0.770) at a median follow-up of 31.5 months, with manageable toxicity profiles and high response rates (ORR 63.1%) (Tereda et al., 2025). Network meta-analyses indicate that this combination may represent the most efficacious first-line treatment approach for the MSI-H subgroup, with potential for enhanced outcomes when combined with epigenetic priming strategies (Chen K. et al., 2024).

Nevertheless, therapeutic challenges persist among immunotherapy-responsive MSI-H patients, as a considerable subset experiences primary or early resistance to anti-PD-1 monotherapy. Experts have observed that trial response rates frequently fall below 50%, thereby underscoring biological heterogeneity and the necessity for additional biomarkers beyond tumor mutational load. This clinical reality creates a strong rationale for biomarker-driven stratification and epigenetic-targeted combinations to address immune-evasion mechanisms such as epigenetic silencing of antigen-presentation pathways (Hyung et al., 2022; Sahin et al., 2024).

The translation of 9p21.3 biology into clinical biomarker applications requires practical implementation strategies. *CDKN2A* methylation analysis can be performed using quantitative pyrosequencing assays that yield single-base-resolution percent methylation values at defined CpG sites and have been validated in large CRC cohorts. Alternative approaches, such as MethyLight-based CIMP panels, offer high-throughput classification of CIMP-high *versus* CIMP-low/negative tumors and can be readily integrated into diagnostic workflows. Integration with existing molecular classification systems offers additional clinical utility, as the combination of MSI status, CIMP classification, and specific gene methylation patterns provides a framework for patient stratification that builds upon established clinical practice (Ahlquist et al., 2008; Bihl et al., 2012).

The documented correlation between CIMP-high status and enhanced immune infiltration provides mechanistic support for combination strategies targeting both epigenetic silencing and immune checkpoint pathways. In a cohort of 133 MSI-H CRCs, CIMP-high tumors exhibited significantly higher densities of CD8^+^ tumor-infiltrating lymphocytes as well as elevated PD-L1 expression (both tumor proportion and combined positive scores) compared to CIMP-low/negative cases, independent of tumor mutational burden, identifying CIMP-high tumors as an immune-“hot” subtype and optimal candidates for immunotherapy combinations. The identification of MSI-H patients with epigenetically “cold” immune phenotypes thus represents a specific population in which the 9p21.3 status should be investigated and epigenetic interventions–such as DNA methyltransferase inhibitors–might restore therapeutic responsiveness to checkpoint inhibition (Kim et al., 2024).

### 2.7 Molecular landscape of MSI-H colorectal cancer

MSI-H colorectal cancer represents a molecularly distinct subtype characterized by dMMR and the accumulation of mutations at repetitive DNA sequences. Understanding the complete molecular architecture of MSI-H CRC is essential for contextualizing 9p21.3-targeted interventions within the broader therapeutic landscape (Chen et al., 2017; Greco et al., 2023). The MSI-H phenotype arises through two principal mechanisms: sporadic epigenetic silencing (predominantly MLH1 promoter hypermethylation, accounting for a majority of MSI-H cases across cohorts) and germline mutations in MMR genes (MLH1, MSH2, MSH6, PMS2) causing Lynch syndrome. MLH1 hypermethylation shows strong association with CIMP-high status and frequently co-occurs with BRAF V600E mutations in sporadic MSI-H tumors, creating a molecular signature distinct from Lynch syndrome-associated cancers. Recent clinical evidence indicates differential immunotherapy outcomes between Lynch syndrome and non-Lynch MSI-H patients, with Lynch-linked cases showing improved progression-free survival under immune checkpoint blockade while both groups derive benefit overall (Kuismanen et al., 2000; Kedrin and Gala, 2015; Seppälä et al., 2015; Chen et al., 2017; Colle et al., 2023).

Beyond classical MMR proteins, several biomarkers refine MSI-H classification and prognostication (Greco et al., 2023). The HSP110 T17 mononucleotide repeat has been proposed as a functional MSI marker; deletions in this sequence correlate with reduced wild-type HSP110 expression and have been reported to associate with improved prognosis in some studies, although large multicenter data indicate it is not a robust prognostic marker and is better considered as a diagnostic adjunct to resolve discordant cases (Kim and Kang, 2014; Kim et al., 2014; Berardinelli et al., 2018; Tachon et al., 2022). Transcriptomic profiling shows MSI-H tumors are enriched for inflammation-related pathways (IL-17 signaling, TNF signaling, chemokine signaling, NFκB activation) and display alterations in metabolic programs compared with microsatellite-stable counterparts (Kibriya et al., 2024). These pathway features correspond to an immune-inflamed microenvironment characterized by dense CD8^+^ tumor-infiltrating lymphocytes, elevated PD-L1 expression, and generally high tumor mutational burden (Kim et al., 2024).

Importantly, rare discordant cases exhibit MSI-H by molecular testing despite proficient MMR protein expression, reflecting mechanisms beyond canonical MMR deficiency. Such cases may involve isolated MSH3 dysfunction–particularly affecting tetranucleotide loci–and can be modulated by IL-6 pathway activity, with functional polymorphisms in the IL-6/gp130 axis (e.g., gp130 + 148G/C) associated with specific MSI patterns. Additionally, inflammation-associated microsatellite alterations observed in non–MSI-H tumors represent a distinct phenomenon linked to chronic inflammation rather than MMR deficiency (Koi et al., 2018; Salar et al., 2024; Xu et al., 2024). The integration of 9p21.3 methylation status with established MSI-H biomarkers offers opportunities for refined patient stratification within broader epigenetic frameworks that include locus-specific methylation at tumor suppressor regions such as *CDKN2A*. In MSI-H cohorts, CIMP-high tumors exhibit significantly higher CD8^+^ TIL densities and PD-L1 expression than CIMP-low/negative cases, independent of tumor mutational burden, highlighting CIMP-high as an attractive subset for epigenetic–immune combination strategies. This multi-dimensional classification - incorporating MSI status, CIMP classification, BRAF mutation, and locus-specific methylation patterns–provides a practical molecular roadmap for precision medicine approaches in colorectal cancer (Silva et al., 2013; Chen et al., 2017; Greco et al., 2023; Kim et al., 2024).

## 3 Epigenetic features of 9p21.3 locus and cancer-associated dysregulation

Epigenetic regulation is a multifaceted process that extends beyond DNA methylation to encompass histone modifications, nucleosome positioning, and higher-order chromatin architecture. In this chapter, we analyze regulation of genes within the 9p21.3 locus at both the “2D” level of local promoter-enhancer states and the “3D” level of genome topology, including long-range interactions and domain organization. This integrated perspective clarifies how locus configuration shapes gene expression and may influence therapeutic responsiveness.

### 3.1 Topological and epigenetic control of gene expression at the 9p21.3 locus

**FIGURE 2:**
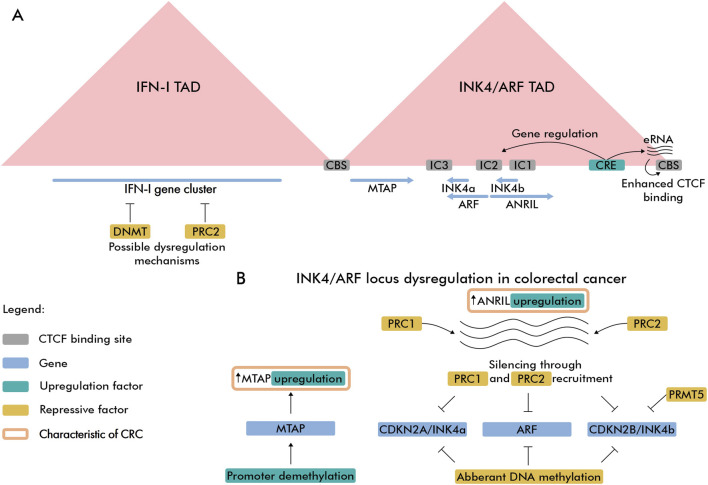
Spatial and regulatory features of 9p21.3 locus in CRC. **(A)** Spatial organization of IFN-I and INK4/ARF TADs. The super-enhancer element (CRE) controlling gene and boundary eRNA expression is highlighted with emerald color. Intergenic CTCF-enriched sites are marked as IC1, IC2 and IC3 (Hirosue et al., 2012). **(B)** CRC-related epigenetic dysregulation features of INK4/ARF locus.

Recent studies revealed that chromatin architecture plays an important role in gene regulation, including 9p21.3 locus. Chromatin in human cells is organized into topologically associating domains (TADs) – submegabase-scale, spatially insulated regions. TAD boundaries are typically demarcated by convergently oriented CTCF binding sites (CBSs) (Szabo et al., 2019; Kabirova et al., 2023). Here, we focus on the chromatin organization of IFN-I and INK4/ARF loci, as more epigenetic data are available for these regions. Hi-C analyses across multiple cell lines demonstrated that *IFN-I* gene cluster and INK4/ARF region are located in separated domains (Figure 2A) (Rao et al., 2014; Islam et al., 2023). Since domain borders restrict inter-TAD interactions between c*is*-regulatory elements (CREs) such as promoters and enhancers, this spatial segregation suggests that the *IFN-I* gene cluster and INK4/ARF region are regulated independently.

The INK4/ARF TAD includes the *CDKN2A*, *ARF*, *CDKN2B*, and *CDKN2B-AS1* genes, along with the *MTAP* gene located near the 5′ domain boundary. Like most TAD boundaries in human cells, INK4/ARF TAD borders are enriched with CTCF binding sites, indicating that CTCF/cohesin-mediated loop extrusion mechanism contributes to formation of this domain. Notably, the 3′TAD boundary contains actively transcribed enhancers that presumably recruit more CTCF proteins to its binding sites and these enhancers have a modest effect on expression of genes within the domain (Islam et al., 2023). TAD also harbors a super-enhancer region located downstream of ANRIL lncRNA gene enriched with active H3K27ac marks. This super-enhancer controls expression of all genes within the domain while simultaneously driving enhancer RNA (eRNA) expression in 3′ boundary CBS. Expression of eRNA can increase chromatin accessibility, thereby facilitating cohesin loading (Li et al., 2023), which reinforces the domain boundary through cohesin/CTCF-dependent loop extrusion. With regard to CTCF, it remains unclear how eRNA expression affects CTCF binding–whether by opening chromatin, directly recruiting CTCF, or both–as recent studies have cast doubt on the RNA-binding capacity of certain chromatin-associated proteins, including CTCF (Guo et al., 2024; Healy et al., 2024). Another *cis-*regulatory element was identified in a region adjacent to *ARF* promoter, which was shown to repress *CDKN2A* expression through looping interaction (Zhang et al., 2019). 3C-experiments revealed that in normal somatic cells, the *CDKN2A*/*ARF*/*CDKN2B* gene cluster adopts a repressive loop conformation (Hirosue et al., 2012), consistent with these genes’ relatively low expression levels in normal cells (Sano et al., 1998). In senescent cells, the loop conformation becomes more relaxed, leading to elevated expression of *CDKN2A* and *CDKN2B* genes and a modest increase in *ARF* expression. These chromatin loop changes reflect differential CTCF binding, which increases in compact conformations and decreases in relaxed states.

In CRC, DNA hypermethylation of the *CDKN2A* promoter correlates with reduced gene expression. This CpG island is an established epigenetic target in CRC, showing up to 20% higher methylation in tumor cells compared to normal colorectal mucosa cells. Particularly, hypermethylation occurs more frequently in MSI-H tumors (23%) than in MSS/MSI-L subtypes (13.4%) (Bihl et al., 2012). Beyond silencing *via* hypermethylation, *CDKN2A* promoter methylation may displace CTCF from its adjacent binding site, potentially enabling heterochromatin spread (Witcher and Emerson, 2009) and loss of loop-mediated interaction with CREs. The *ARF* gene is frequently downregulated in CRC, with promoter methylation (observed in 29%–33% of patients) correlating with poor prognosis (Dominguez et al., 2003; Nilsson et al., 2012). Notably, in colon carcinoma *ARF* promoter methylation often occurs independently of *CDKN2A*. In 52% of cases *ARF* was methylated while *CDKN2A* remained unmethylated, suggesting distinct regulatory mechanisms (Esteller et al., 2000). *CDKN2B* is also a frequent target for DNA hypermethylation in CRC. In 89% of cases in Egyptian patients, downregulation of *CDKN2B* was associated with its promoter hypermethylation (Abdel-Rahman et al., 2014). Moreover, *CDKN2B* was identified as a target of EZH2 histone methyltransferase activity in CRC (Yang et al., 2021). EZH2 was also shown to associate with PRMT5 arginine methyltransferase, resulting in the deposition of repressive histone marks at the *CDKN2B* promoter–H3K27me3 (PRC2) and H4R3me2s/H3R8me2s (PRMT5). Importantly, treatment with EZH2 and PRMT5 inhibitors activated *CDKN2B* transcription and attenuated CRC cell growth, demonstrating potential therapeutic relevance.

ANRIL (Antisense Noncoding RNA in the INK4 Locus, *CDKN2B-AS1*) is a long noncoding RNA transcribed from the bidirectional promoter it shares with the *ARF* gene. The *CDKN2B-AS1* gene spans the entire *CDKN2B* gene and is transcribed in the antisense direction. This lncRNA is suspected to recruit PRC1 and PRC2 repressive protein complexes, leading to accumulation of H3K27me3 mark and silencing of adjacent genes (Yap et al., 2010). In colon carcinomas, ANRIL overexpression was observed in invasive tumors (12%) and was higher in carcinomas at metastatic stage (16%) (Drak Alsibai et al., 2019). ANRIL epigenetically regulates the INK4/ARF locus by recruiting Polycomb complexes: it binds PRC1/CBX7 and PRC2 components to deposit repressive H3K27me3 and silence *CDKN2A/CDKN2B*, thereby constraining p16^INK4a^/p14^ARF^/p15^INK4b^ expression and senescence checkpoints (Yap et al., 2010; Kotake et al., 2011). During oncogene-induced senescence, circular ANRIL isoforms can switch roles and facilitate INK4 activation by sequestering Polycomb factors, highlighting isoform- and context-specific epigenetic control at 9p21.3 (Muniz et al., 2020).

Collectively, tumor suppressor genes in the 9p21.3 locus exhibit diverse expression regulation mechanisms in CRC, including DNA methylation, Polycomb-mediated suppression, and ANRIL-dependent silencing (Figure 2B). Interestingly, the *MTAP* gene is frequently overexpressed in CRC due to promoter hypomethylation (Dou et al., 2009; Zhong et al., 2018), in contrast to hypermethylation of tumor suppressor genes at the 9p21.3 locus.

Type I IFNs are known to modulate chromatin architecture and accessibility at target loci (Platanitis et al., 2022), yet the 3D organization of the *IFN-I* gene cluster remains to be defined. Hi-C analyses confirm strong insulation of both INK4/ARF and IFNs TADs across cell types, though HUVEC cells exhibit unique long-range contacts between the IFNA21 downstream region and an ANRIL downstream enhancer (Harismendy et al., 2011). While *IFN-I* cluster regulation in CRC requires further study, these genes are subject to PRC2-mediated silencing in breast cancer–a phenomenon correlated with diminished antitumor immune responses (Hong et al., 2022). Additionally, single CpG methylation has been reported to silence *type I IFNs* expression, potentially through an indirect mechanism that disrupts IRF3 binding to promoter regions. Targeted demethylation of the *Ifnβ1* promoter in mice increased its expression, demonstrating DNA methylation’s role in regulating *Ifnβ1* transcription (Gao et al., 2021). Thus, *type I IFNs* expression is regulated by both PRC2-mediated silencing and DNA methylation, making these mechanisms potential therapeutic targets. Interferon signaling activity also depends on epigenetic modifications of RNA, in particular m5C. One of the RNA methyltransferases that provides m5C modification is NSUN2, which is highly expressed in CRC tumors. NSUN2 maintains m5C modifications, which in particular leads to the stabilization of TREX2, a protein with exonuclease activity. TREX2 degrades double-stranded DNA and thus inhibits the cGAS-STING pathway, type I IFNs expression, and promotes tumor growth (Sun et al., 2025).

### 3.2 Therapeutic targeting of epigenetic dysregulation

Recent studies have demonstrated the therapeutic potential of targeting epigenetic regulators within the 9p21.3 locus (Supplementary Table S1). EZH2 inhibition, particularly with tazemetostat, has shown promise in preclinical models for restoring *CDKN2B* expression in CRC (Yang et al., 2021). Tazemetostat, a selective EZH2 inhibitor approved by the FDA for epithelioid sarcoma and relapsed/refractory follicular lymphoma (Hoy, 2020; Straining and Eighmy, 2022; Orleni and Beumer, 2024), demonstrated transcriptional activation of *CDKN2B* and attenuated CRC cell growth when combined with PRMT5 inhibitors in preclinical studies, indicating the potential for dual methyltransferase targeting. However, recent preclinical evidence indicates that EZH2 inhibition may paradoxically enhance PD-L1 protein stability through USP22-mediated deubiquitination in CRC, potentially creating immune suppressive effects that could be overcome through combination with immunotherapy (Huang J. et al., 2024). The CAIRE phase II trial evaluated tazemetostat plus durvalumab in patients with advanced microsatellite stable CRC, achieving the primary endpoint with a disease control rate of 35.3%, with circulating H3K27me3-modified nucleosomes serving as potential pharmacodynamic biomarkers for EZH2 target engagement (Palmieri et al., 2023).

Early clinical translation of DNMT inhibitors in CRC has been largely disappointing, with recurrent pharmacodynamic and trial-design limitations that likely explain modest/absent activity in pMMR/MSS disease. In the pre-operative DECO trial (NCT01882660), decitabine 25 mg/m^2^ on two consecutive days led only to a small but statistically significant drop in tumor LINE1 methylation (71.2% -> 67.2%, *p* = 0.0075) and did not change methylation/expression of WNT target genes or induce ERV/interferon programs, prompting premature closure after 10 treated patients (Linnekamp et al., 2021). In METADUR (NCT02811497), oral CC-486 plus durvalumab produced no objective responses (DCR 7.1%, median PFS 1.9 months) and showed minimal tumor demethylation with absent viral-mimicry signaling; PBMC LINE-1 demethylation was typically <10% (overall maximum 19.9%; maximum 13.8% in MSS-CRC), consistent with insufficient on-target activity *in vivo* (Taylor et al., 2020). Similarly, pembrolizumab plus low-dose subcutaneous azacitidine (100 mg days 1–5; NCT02260440) achieved ORR 3% and median PFS 1.9 months despite evidence of on-treatment tumor demethylation (10/15 paired biopsies, 67%), suggesting that biochemical demethylation alone - without optimal dose/schedule and biomarker enrichment - may be insufficient to consistently generate clinically meaningful immune priming in refractory mCRC (Kuang et al., 2022). Outside ICI combinations, a CIMP-enriched DNMTi/chemotherapy strategy (azacitidine + CAPOX; NCT01193517) likewise yielded no objective responses, although stable disease occurred in 65% (17/26; median duration 4.5 months), underscoring that patient selection by broad methylation phenotype alone does not guarantee antitumor responses (Overman et al., 2016). In contrast, the planned phase II NCT07007767 in heavily pretreated pMMR/MSS CRC evaluates decitabine “priming” combined with sintilimab and bevacizumab, attempting to pair epigenetic modulation with a backbone expected to be more permissive for immune infiltration; notably, locus-level selection (e.g., intact, hypermethylated 9p21.3) is not currently part of eligibility and should be considered for future iterations (Zhang, 2025).

Updated clinical evidence supports the immune-independent augmentation of chemotherapy by low-dose decitabine. The sequential combination of gemcitabine followed by decitabine has demonstrated synergistic effects through ribonucleotide reductase inhibition, providing a promising paradigm for enhancing chemotherapy efficacy while potentially reducing toxicity (Gutierrez et al., 2022). This approach differs from traditional DNA-demethylating pretreatment strategies and may be particularly relevant for tumors with epigenetically silenced 9p21.3 genes.

## 4 The role of 9p21.3 locus genes in the senescence in cancer

Senescence is a non-proliferative but viable state of a cell, usually induced by various stress factors. The senescent state is characterized by prolonged and usually irreversible cell cycle arrest with altered metabolism, secretory features, and macromolecular damage (Gorgoulis et al., 2019; Chambers et al., 2021).

Cell cycle arrest in senescence is usually irreversible, but cell cycle re-entry may occur under certain circumstances, especially in tumor cells (Patel et al., 2016; Le Duff et al., 2018; Guillon et al., 2019). Senescent cells are characterized by enlarged, flattened morphology and nuclear abnormalities–including an enlarged nucleus and loss of lamin B1, sustained expression of *CDKN2A* (p16^INK4a^) and/or *CDKN1A* (p21^Cip1^), heterochromatin remodeling (senescence-associated heterochromatin foci, SAHF), mitochondrial dysfunction, metabolic rewiring, and increased lysosomal β-galactosidase activity (SA-β-gal). A defining hallmark is the senescence-associated secretory phenotype (SASP) – a coordinated program of cytokines (e.g., IL-6, IL-8), chemokines, growth factors, and matrix-remodeling enzymes–that actively reshapes the tumor microenvironment and is therefore highly consequential for tumorigenesis, immune surveillance, and therapeutic response (Schmitt et al., 2022; Wang et al., 2022).

Historically, senescence of cancer cells was viewed as a barrier to malignant progression (Haferkamp et al., 2009), but more recent work shows that senescent tumor cells can also promote disease *via* SASP-driven protumorigenic effects (Kim et al., 2017; Choi et al., 2021). Both senescent cancer cells and non-transformed senescent cells residing in the tumor microenvironment can affect treatment outcomes in CRC (Bogdanova et al., 2024; Pukhalskaia et al., 2024). Multiple modalities–including classical cytotoxics, targeted agents, and immunotherapies–can induce therapy-induced senescence (TIS) (Wang et al., 2022). Similarly, demethylating therapies can trigger this process through reactivation of cell cycle inhibitors at the 9p21.3 locus. Moreover, cellular senescence can be induced by oncogenic activation, a process known as oncogene-induced senescence (OIS) (Bartkova et al., 2006).

The following sections analyze the contributions of the 9p21.3 locus to the biology of senescence in cancer, with an emphasis on CRC, while highlighting the complementary functions described in other tumor types that suggest testable hypotheses regarding their relevance in CRC.

### 4.1 The regulation of *CDKN2A/B* in senescence

The *CDKN2A/B* gene products, p16^INK4a^, p15^INK4b^, and p14^ARF^, induce cell cycle arrest that could become permanent if their expression persists, provoking the formation of a senescent phenotype.

Interestingly, the expression of the *CDKN2A/B* genes, which are major regulators of senescence, is controlled by neighboring genes. During normal cell cycle progression, p16^INK4a^, p15^INK4b^, and p14^ARF^ are inhibited by Polycomb proteins, which are recruited by the long noncoding RNA ANRIL. Thus, ANRIL prevents cell cycle arrest (Muniz et al., 2020). A 2016 study showed that ANRIL is overexpressed in CRC and that it positively influences cell proliferation (Naemura et al., 2016). In this case, inhibition of ANRIL resulted in decreased proliferation. It was also noted that ANRIL inhibition did not result in activation of *CDKN2A/B* expression. Increased ANRIL expression in CRC was also observed in another study, with it being highest at the metastatic stage (Drak Alsibai et al., 2019). A later study by Lisa Muniz et al. showed that ANRIL can act as an activator of *CDKN2A/B* expression in circular isoforms during OIS initiation in fibroblast cultures (Muniz et al., 2020). The researchers suggest that this effect is observed because circular ANRIL can bind to suppressor proteins Polycomb and thus make the chromatin conformation open. Another long non-coding RNA that increases *CDKN2A/B* expression including in CRC cells is P14AS, which can also bind to the Polycomb protein (Li Z. et al., 2022).

Methyltransferase METTL3, which increases the stability of *CDKN2B* mRNA, is also involved in maintaining the formation of senescent cells in CRC (Chen Z. et al., 2024). It has been shown that there is a positive correlation between *CDKN2B* expression and tumor infiltration by tumor-associated macrophages (Chen Z. et al., 2024). Thus, increased *CDKN2B* expression not only leads to the formation of senescent cells, but also affects the tumor microenvironment, making it immunosuppressive.

### 4.2 The role of type I IFNs in senescence

When DNA double-strand breaks occur, one of the key proteins that activates is ATM kinase which triggers a cascade of reactions leading to phosphorylation and activation of p53, a transcription factor that is one of the main tumor suppressors. p53 triggers the expression of the cyclin-dependent kinase inhibitor *CDKN1А* (Schmitt et al., 2022). However, this is not the only effect of ATM activation. It has been shown both *in vitro* and *in vivo* that ATM can also activate IRF3 which triggers the expression of *IFNβ1*. The resulting IFNβ increases the expression of *CDKN1А* and *CDKN2A/B*, thereby contributing to the establishment of senescence (Yu et al., 2015). Also, as noted above, other studies highlight cell cycle arrest and senescence induction by type I IFNs *via CDKN1A*, *CDKN1B*, and *CDKN2B* (Katayama et al., 2007; Musella et al., 2021; Mödl et al., 2023). Type I IFNs play a significant role in the development of senescence. Yulia V. Katlinskaya et al. demonstrated that inhibition of interferon signaling resulted in suppression of senescence and the development of melanoma (Katlinskaya et al., 2016).

Several studies have examined the hypothesis that cellular senescence is a defense against viruses (Reddel, 2010; Yu et al., 2015). There is a correlation between the amount of type I IFNsproduced and the number of double-strand breaks that can occur as a result of infection by genome integrating viruses (Yu et al., 2015; Ryan et al., 2016). So, the increase in type I IFNs synthesis in response to DNA damage may initially be associated with antiviral protection. The activation of type I IFNs synthesis may be also associated with the transcriptional depression of the LINE-1 retrotransposon element observed during senescence which was shown in human fibroblasts (Cecco et al., 2019).

Type I IFNs are also a component of the SASP secretome (Birch and Gil, 2020). Type I IFNs may be involved in maintaining chronic inflammation in the tumors (Cecco et al., 2019; Wang D. et al., 2024). Conversely, it has been shown that type I IFNs secreted by SASP can promote the destruction of senescent cells in the tumor by attracting NK cells since type I IFNs increase the expression of NK cell receptor ligands (Katlinskaya et al., 2015).

Thus, type I IFNs are essential for promoting senescence and maintaining their secretory phenotype. However, their role as a component of the SASP in tumors is not clear. On the one hand, type I IFNs can maintain chronic inflammation, and on the other hand, they can promote the removal of tumor cells.

### 4.3 Senescence-targeted therapeutic strategies

The role of 9p21.3-encoded proteins p16^INK4a^ and p15^INK4b^ in cellular senescence creates potential therapeutic opportunities through senolytic agents. These proteins are central regulators of senescence induction, and their methylation-mediated silencing may affect therapy-induced senescence responses (Jung et al., 2020).

A recent study in 2025 showed that cancer cells with decreased DNA methylation enter cellular senescence. Experiments with xenografts show that tumor cells can be induced to undergo senescence *in vivo* by reducing DNA methylation (Chen et al., 2025). These results highlight the importance of epigenetic changes and reduced DNA methylation in cancer cells, which may have practical implications for future therapeutic approaches.

Preclinical studies have explored senolytic agents such as quercetin, navitoclax, and fisetin in CRC models (Russo et al., 2023; Bogdanova et al., 2024). However, clinical data remain limited, and recent evidence raises safety concerns about potential tumor-promoting effects of certain senolytic agents (Wyld et al., 2020). In CRC specifically, some senolytic agents may differentially affect SASP components, potentially promoting epithelial-mesenchymal transition and tumor progression (Gallegos et al., 2023).

Emerging evidence supports rational combination strategies targeting multiple epigenetic pathways within the 9p21.3 locus. Recent preclinical studies demonstrate that combining DNA methyltransferase inhibitors with histone methyltransferase inhibitors can overcome adaptive resistance mechanisms. In BRAF^V600E^ CRC models, 5-azacitidine treatment induced compensatory H3K27 trimethylation at demethylated genomic regions, but combination with EZH2 or RNF2 inhibitors showed additive growth inhibitory effects. This finding suggests that adaptive interactions between epigenetic modifiers may limit single-agent efficacy and supports the development of combinatorial epigenetic therapeutic strategies (Lee et al., 2024). The concept of sequential epigenetic priming followed by targeted therapy represents a promising approach for restoring 9p21.3 tumor suppressor function in CRC.

Current clinical trials of senolytic agents extends beyond age-related diseases into cancer, yet specific studies remain sparse and are rarely designed to explicitly test senolytic mechanisms. For instance, navitoclax appears only in a trametinib combination cohort without senescence biomarker readouts, while isoquercetin has been evaluated for VTE prevention rather than senolysis [National Cancer Institute (NCI), 2025]. Trials with dasatinib in CRC have focused on its role as a SRC kinase inhibitor rather than leveraging its senolytic potential, and designs with dasatinib + quercetin or others senolytic compounds specifically in CRC are lacking, while senescence-focused biomarker stratification (e.g., p16^INK4a^/SASP panels; linkage to 9p21.3 methylation) is rarely incorporated [National Cancer Institute (NCI), 2014]. Preclinical investigations reveal significant therapeutic potential through “one-two punch” approaches that first induce senescence followed by senolytic elimination (Khosla, 2024; Czajkowski et al., 2025; López et al., 2025; St. Jude Children’s Research Hospital, 2025).

However, clinical translation of senolytic strategies in colorectal cancer faces significant challenges and remains largely experimental. Most available evidence derives from preclinical models, which may not fully recapitulate the complexity of human CRC biology. Furthermore, recent preclinical studies have revealed concerning safety signals, including differential effects of senolytic agents on SASP components and potential promotion of epithelial-mesenchymal transition. For instance, while navitoclax effectively reduces IL-6 secretion in senescent CAFs, the dasatinib-quercetin combination paradoxically increases IL-6 levels and promotes tumor cell migration in colorectal cancer models (Bogdanova et al., 2024). These findings underscore the necessity of tailoring senolytic timing and combinations to modulate SASP appropriately and emphasize the need for more careful evaluation of senolytic strategies before clinical implementation.

Recent clinical trials provide compelling evidence for senolytic efficacy in cancer-adjacent applications. The SENSURV trial (NCT04733534) at St. Jude Children’s Research Hospital represents a landmark Phase 2 study evaluating dasatinib plus quercetin *versus* fisetin monotherapy in 110 adult survivors of childhood cancer. This trial specifically measures senescent cell abundance (primary outcome: p16^INK4a^) and frailty markers, establishing crucial biomarkers for senolytic efficacy assessment (St. Jude Children’s Research Hospital, 2025). The Mayo Clinic skeletal health study (NCT04313634) further validates senolytic mechanisms, administering intermittent dasatinib (100 mg) plus quercetin (1,000 mg) cycles to elderly postmenopausal women, demonstrating measurable reductions in senescent cell burden and inflammatory markers (Khosla, 2024). Most significantly, the Mayo Clinic glioma trial (NCT07025226) represents the first sequential senolytic cancer treatment protocol, employing dasatinib-quercetin combinations followed by fisetin and temozolomide in previously treated patients (Mayo Clinic, 2025). Additionally, a Phase 2 triple-negative breast cancer trial (NCT06355037) is currently recruiting patients to evaluate dasatinib (50 mg) plus quercetin (1000 mg) combined with chemotherapy to reverse treatment resistance, based on preclinical evidence showing effective elimination of chemotherapy-induced senescent fibroblasts (Shao, 2024).

Moreover, a “one-two punch” approach combining talazoparib with palbociclib induces robust therapy-induced senescence in CRC xenografts, and subsequent PD-L1 blockade eradicates senescent cells to deliver significant survival benefits in immunocompetent mice. Comparable senolytic selectivity extends beyond CRC: in glioblastoma models, navitoclax reduces viability of senescent cells by over 60% with minimal impact on proliferating cells, demonstrating BCL-XL dependency across radiated and TMZ-treated human glioma cell lines (Rahman et al., 2022). In lung adenocarcinoma A549 cells, therapy-induced senescence similarly confers high sensitivity to BCL-XL-targeting agents such as A1331852, with marked senolytic selectivity observed across multiple TIS phenotypes (López et al., 2025). These findings underscore the necessity of tailoring senolytic timing and combinations to modulate SASP appropriately and maximize anti-tumor efficacy (Wang et al., 2023; Czajkowski et al., 2025) and emphasizing the need for more careful evaluation of senolytic strategies before clinical implementation. Similarly, venetoclax (ABT-199), a navitoclax derivative, demonstrated senolytic efficacy in APTKA orthotopic rectal cancer models, where venetoclax treatment significantly reduced tumor burden, suppressed invasive growth, and prevented liver metastasis formation when combined with radiotherapy. The combination also led to decreased collagen deposition, reduced DCN + fibroblast numbers, and enhanced CD8^+^ T cell infiltration. However, venetoclax exhibited dual effects, as it also directly impaired organoid growth *ex vivo*, particularly in non-irradiated conditions, suggesting that improved therapeutic responses may result from both senolytic activity and direct pro-apoptic effects on tumor cells (Nicolas et al., 2022). Nevertheless, proof-of-concept studies continue to demonstrate therapeutic potential.

### 4.4 Integrated biomarker-guided therapeutic algorithm

To translate the complex landscape of 9p21.3 alterations into clinical utility, we propose a stratified therapeutic algorithm based on the structural and epigenetic status of the locus (Supplementary Table S2). This framework distinguishes between irreversible genomic loss (deletions) and reversible epigenetic silencing (methylation), integrating recent advances in synthetic lethality and immunotherapy (Song and Yang, 2025; Subramaniam et al., 2025).

#### 4.4.1 Structural deletion of 9p21.3 (*MTAP*-deficient/*type I IFNs*-null)

Tumors harbouring homozygous deletions of 9p21.3 invariably lose *MTAP* and frequently the *IFN-I* gene cluster alongside *CDKN2A/B*. These tumors are characteristically ‘immune-cold’ due to the loss of type I interferon signaling, rendering them potentially resistant to immune checkpoint blockade monotherapy. Therapeutic strategy: the primary vulnerability is metabolic. *MTAP* loss creates a synthetic lethal dependence on the PRMT5-MAT2A axis (Gounder et al., 2025). MTA-cooperative PRMT5 inhibitors (for example, MRTX1719) and MAT2a inhibitors (Cann et al., 2023; Helwick, 2024; Andre et al., 2025) (e.g., IDE397) have shown efficacy in recent years in solid tumors (Cann et al., 2023; Helwick, 2024; Andre et al., 2025). Combination approach to overcome the “cold” immune microenvironment, combining PRMT5 inhibitors with immune checkpoint blockade is promising. Emerging data suggest that PRMT5 inhibition can sensitize tumor cells to T-cell mediated cytotoxicity and downregulate immune-exclusionary pathways (e.g., PI3K), offering a rationale for combination even in the context of compromised IFN signaling (Chen S. et al., 2024; Bartosik et al., 2025; Song and Yang, 2025).

#### 4.4.2 Intact 9p21.3 with hypermethylation (CIMP-H/*CDKN2A*-silenced)

This subset retains the genetic code for *CDKN2A* and *type I IFNs*, but suppresses them epigenetically. These tumors frequently overlap with MSI-H phenotype and high tumor mutational burden (Reyila et al., 2025). Therapeutic strategy: reversal of silencing is key. DNMTi like azacitidine or decitabine can demethylate the *CDKN2A* promoter, restoring p16^INK4a^ expression and re-activiting the viral mimicry dsRNA pathways (Roulois et al., 2015). Demethylation therapy also restore the expression of *type I IFNs*, which can contribute to the tumor becoming “hot”. Combinational approaches are: a) Immune checkpoint inhibitors-responsive: standard of care involves anti-PD-1/CTLA-4 regimens (e.g., nivolumab + ipilimumab), which demonstrated superior progression-free survival in recent Phase III trials (CheckMate 8HW) (Cann et al., 2023; Helwick, 2024; Andre et al., 2025). Adding DNMTi could deepen responses in refractory cases, enhancing antigen presentation senescence-targeted: re-expression of p16^INK4a^ *via* DNMTi acts as a “senogenic” stimulus, arresting tumor cells (Huang K. C.-Y. et al., 2024; 2025). This created a therapeutic window for “senolytic” agents (e.g., BCL-2/xL inhibitors) to selectively eliminate the arrested senescent cells - a sequential “one-two punch” strategy (Wang Y. et al., 2024; Lam et al., 2025; Tajudeen et al., 2025).

#### 4.4.3 Intact 9p21.3 with low methylation (CIMP-L/MSS)

These tumors generally express functional MTAP and basal levels of p16^INK4a^, but lack the high immunogenicity of MSI-H tumors. Therapeutic strategy: the focus shifts to inducing immunogenicity and senescence. Сombination approach, standard chemotherapy or CDK4/6 inhibitors can define the senogenic step, followed by senolytic clearance (Wang Y. et al., 2024; Subramaniam et al., 2025).

## 5 Conclusion

A better understanding of genetic and epigenetic regulatory mechanisms, particularly cancer-specific changes, will facilitate the study of their potential clinical applications as biomarkers or therapeutic targets in colorectal cancer. To date, accumulated data provide compelling evidence that epigenetic dysregulation is an important factor in colorectal cancer progression and therapeutic resistance development. In particular, DNA hypermethylation of tumor suppressor gene promoter regions is associated with a poor prognosis, an increased risk of relapse and metastasis, and a reduced effectiveness of standard therapeutic approaches. *CDKN2A* hypermethylation is the most indicative in this regard and has repeatedly been shown to be associated with poor survival for patients.

Locus 9p21.3 is a unique gene cluster combining cell cycle and senescence control genes (*CDKN2A*/*CDKN2B*), immune response modulators (*IFN-I* gene cluster), and metabolic factors (*MTAP*, *MLLT3*). Disruption of the expression of these genes due to hypermethylation or deletion can lead to the loss of antitumor checkpoints, reduced tumor immunogenicity, and resistance to therapy. Therefore, status of the 9p21.3 locus is a promising biomarker for patient stratification and therapy selection.

This approach enables us to stratify patients for whom the epigenetic reactivation of the locus can restore cell cycle control, enhance the antitumor immune response, and improve the effectiveness of follow-up treatment. Concurrently, the dual role of type I IFNs and senescence in tumors underscores the necessity of strictly controlling the timing and duration of demethylating therapy and considering the use of senolytic agents to mitigate the adverse effects of chronic senescence.

On the other hand, for cases involving the deletion of 9p21.3, an alternative approach based on exploiting the synthetic lethality between MTAP deficiency and the use of PRMT5/MT2A inhibitors would benefit this cohort of CRC patients.

It is important to note that all clinical studies evaluating epigenetic therapy for colorectal cancer were conducted on patients with advanced stages of the disease for whom other treatments had been ineffective. Under these conditions, the use of demethylating drugs as monotherapy predictably demonstrated limited effectiveness. Additionally, accumulated data suggest the potential of using demethylating agents not as standalone treatments but as tools for epigenetic “priming” that increase tumor sensitivity to chemotherapy, radiation, and immunotherapy (Jung et al., 2020).

In conclusion, DNA methylation biomarkers are widely associated with prognosis and survival. However, their applications as biomarkers that could alter current treatment strategies are limited. Nevertheless, we believe that the biomarkers presented here warrant further evaluation in prospective studies due to the highly promising preliminary data on their utility.

## References

[B1] Abdel-Rahman W. M. Nieminen T. T. Shoman S. Eissa S. Peltomaki P. (2014). Loss of p15INK^4^b expression in colorectal cancer is linked to ethnic origin. Asian Pac J. Cancer Prev. 15, 2083–2087. 10.7314/apjcp.2014.15.5.2083 24716938

[B2] Ahlquist T. Lind G. E. Costa V. L. Meling G. I. Vatn M. Hoff G. S. (2008). Gene methylation profiles of normal mucosa, and benign and malignant colorectal tumors identify early onset markers. Mol. Cancer 7, 94. 10.1186/1476-4598-7-94 19117505 PMC2639620

[B208] Alum E. U. Izah S. C. Uti D. E. Ugwu O. P.-C. Betiang P. A. Basajja M. (2025). Targeting cellular senescence for healthy aging: advances in senolytics and senomorphics. Drug Des. Develop. Ther. 19, 8489–8522. 10.2147/DDDT.S543211 40994903 PMC12456441

[B3] Andre T. Elez E. Van Cutsem E. Jensen L. H. Bennouna J. Mendez G. (2024). Nivolumab (NIVO) plus ipilimumab (IPI) vs chemotherapy (chemo) as first-line (1L) treatment for microsatellite instability-high/mismatch repair-deficient (MSI-H/dMMR) metastatic colorectal cancer (mCRC): first results of the CheckMate 8HW study. J. Clin. Oncol. 42, LBA768. 10.1200/JCO.2024.42.3_suppl.LBA768

[B4] Andre T. Elez E. Lenz H.-J. Jensen L. H. Touchefeu Y. Van Cutsem E. (2025). First results of nivolumab (NIVO) plus ipilimumab (IPI) vs NIVO monotherapy for microsatellite instability-high/mismatch repair-deficient (MSI-H/dMMR) metastatic colorectal cancer (mCRC) from CheckMate 8HW. J. Clin. Oncol. 43, LBA143. 10.1200/JCO.2025.43.4_suppl.LBA143

[B5] Barekatain Y. Ackroyd J. J. Yan V. C. Khadka S. Wang L. Chen K.-C. (2021). Homozygous MTAP deletion in primary human glioblastoma is not associated with elevation of methylthioadenosine. Nat. Commun. 12, 4228. 10.1038/s41467-021-24240-3 34244484 PMC8270912

[B6] Barriga F. M. Tsanov K. M. Ho Y.-J. Sohail N. Zhang A. Baslan T. (2022). MACHETE identifies interferon-encompassing chromosome 9p21.3 deletions as mediators of immune evasion and metastasis. Nat. Cancer 3, 1367–1385. 10.1038/s43018-022-00443-5 36344707 PMC9701143

[B7] Bartkova J. Rezaei N. Liontos M. Karakaidos P. Kletsas D. Issaeva N. (2006). Oncogene-induced senescence is part of the tumorigenesis barrier imposed by DNA damage checkpoints. Nature 444, 633–637. 10.1038/nature05268 17136093

[B8] Bartosik A. Radzimierski A. Bobowska A. Więckowska A. Kuś K. Faber J. (2025). Abstract 4231: preclinical candidate RVU305, an MTA-cooperative PRMT5 inhibitor, shows activity in MTAP-deleted tumors resistant to immune checkpoint treatment. Cancer Res. 85, 4231. 10.1158/1538-7445.AM2025-4231

[B9] Bedard G. T. Gilaj N. Peregrina K. Brew I. Tosti E. Shaffer K. (2023). Combined inhibition of MTAP and MAT2a mimics synthetic lethality in tumor models *via* PRMT5 inhibition. J. Biol. Chem. 300, 105492. 10.1016/j.jbc.2023.105492 38000655 PMC10770533

[B10] Belhadj S. Terradas M. Munoz-Torres P. M. Aiza G. Navarro M. Capellá G. (2020). Candidate genes for hereditary colorectal cancer: mutational screening and systematic review. Hum. Mutat. 41, 1563–1576. 10.1002/humu.24057 32449991

[B11] Berardinelli G. N. Scapulatempo-Neto C. Durães R. Antônio de Oliveira M. Guimarães D. Reis R. M. (2018). Advantage of HSP110 (T17) marker inclusion for microsatellite instability (MSI) detection in colorectal cancer patients. Oncotarget 9, 28691–28701. 10.18632/oncotarget.25611 29983889 PMC6033349

[B12] Bihl M. P. Foerster A. Lugli A. Zlobec I. (2012). Characterization of CDKN2A(p16) methylation and impact in colorectal cancer: systematic analysis using pyrosequencing. J. Transl. Med. 10, 173. 10.1186/1479-5876-10-173 22925370 PMC3479016

[B13] Birch J. Gil J. (2020). Senescence and the SASP: many therapeutic avenues. Genes Dev. 34, 1565–1576. 10.1101/gad.343129.120 33262144 PMC7706700

[B14] Bogdanova D. A. Kolosova E. D. Pukhalskaia T. V. Levchuk K. A. Demidov O. N. Belotserkovskaya E. V. (2024). The differential effect of senolytics on SASP cytokine secretion and regulation of EMT by CAFs. Int. J. Mol. Sci. 25, 4031. 10.3390/ijms25074031 38612842 PMC11012227

[B15] Brand F. Förster A. Christians A. Bucher M. Thomé C. M. Raab M. S. (2020). FOCAD loss impacts microtubule assembly, G2/M progression and patient survival in astrocytic gliomas. Acta Neuropathol. 139, 175–192. 10.1007/s00401-019-02067-z 31473790

[B16] Bray F. Laversanne M. Sung H. Ferlay J. Siegel R. L. Soerjomataram I. (2024). Global cancer statistics 2022: GLOBOCAN estimates of incidence and mortality worldwide for 36 cancers in 185 countries. CA Cancer J. Clin. 74, 229–263. 10.3322/caac.21834 38572751

[B17] Cann C. G. LaPelusa M. B. Cimino S. K. Eng C. (2023). Molecular and genetic targets within metastatic colorectal cancer and associated novel treatment advancements. Front. Oncol. 13, 1176950. 10.3389/fonc.2023.1176950 37409250 PMC10319053

[B18] Cao Q. Tian Y. Deng Z. Yang F. Chen E. (2024). Epigenetic alteration in colorectal cancer: potential diagnostic and prognostic implications. Int. J. Mol. Sci. 25, 3358. 10.3390/ijms25063358 38542332 PMC10969857

[B19] Casak S. J. Marcus L. Fashoyin-Aje L. Mushti S. L. Cheng J. Shen Y.-L. (2021). FDA Approval Summary: pembrolizumab for the first-line treatment of patients with MSI-H/dMMR advanced unresectable or metastatic colorectal carcinoma. Clin. Cancer Res. 27, 4680–4684. 10.1158/1078-0432.CCR-21-0557 33846198 PMC8416693

[B20] Cecco M. D. Ito T. Petrashen A. P. Elias A. E. Skvir N. J. Criscione S. W. (2019). LINE-1 derepression in senescent cells triggers interferon and inflammaging. Nature 566, 73–78. 10.1038/s41586-018-0784-9 30728521 PMC6519963

[B21] Chambers C. R. Ritchie S. Pereira B. A. Timpson P. (2021). Overcoming the senescence‐associated secretory phenotype (SASP): a complex mechanism of resistance in the treatment of cancer. Mol. Oncol. 15, 3242–3255. 10.1002/1878-0261.13042 34137158 PMC8637570

[B22] Chen W. Swanson B. J. Frankel W. L. (2017). Molecular genetics of microsatellite-unstable colorectal cancer for pathologists. Diagn. Pathol. 12, 24. 10.1186/s13000-017-0613-8 28259170 PMC5336657

[B23] Chen K. Chen W. Yue R. Zhu D. Cui S. Zhang X. (2024a). Evaluation of the efficacy and safety of first- and second-line immunotherapy in patients with metastatic colorectal cancer: a systematic review and network meta-analysis based on randomized controlled trials. Front. Immunol. 15, 1439624. 10.3389/fimmu.2024.1439624 39359729 PMC11444977

[B24] Chen S. Hou J. Jaffery R. Guerrero A. Fu R. Shi L. (2024b). MTA-cooperative PRMT5 inhibitors enhance T cell-mediated antitumor activity in MTAP-loss tumors. J. Immunother. Cancer 12, e009600. 10.1136/jitc-2024-009600 39313308 PMC11418539

[B25] Chen Z. Zhou J. Wu Y. Chen F. Li J. Tao L. (2024c). METTL3 promotes cellular senescence of colorectal cancer *via* modulation of CDKN2B transcription and mRNA stability. Oncogene 43, 976–991. 10.1038/s41388-024-02956-y 38361047

[B26] Chen X. Yamaguchi K. Rodgers B. Goehrig D. Vindrieux D. Lahaye X. (2025). DNA methylation protects cancer cells against senescence. Nat. Commun. 16, 5901. 10.1038/s41467-025-61157-7 40595593 PMC12216915

[B27] Cheng X. Yang F. Li Y. Cao Y. Zhang M. Ji J. (2024). The crosstalk role of CDKN2A between tumor progression and cuproptosis resistance in colorectal cancer. Aging (Albany NY) 16, 10512–10538. 10.18632/aging.205945 38888512 PMC11236303

[B28] Chiappinelli K. B. Strissel P. L. Desrichard A. Li H. Henke C. Akman B. (2015). Inhibiting DNA methylation causes an interferon response in cancer *via* dsRNA including endogenous retroviruses. Cell 162, 974–986. 10.1016/j.cell.2015.07.011 26317466 PMC4556003

[B29] Chin E. N. Sulpizio A. Lairson L. L. (2023). Targeting STING to promote antitumor immunity. Trends Cell Biol. 33, 189–203. 10.1016/j.tcb.2022.06.010 35931610

[B30] Choi Y. W. Kim Y. H. Oh S. Y. Suh K. W. Kim Y. Lee G. (2021). Senescent tumor cells build a cytokine shield in colorectal cancer. Adv. Sci. (Weinh) 8, 2002497. 10.1002/advs.202002497 33643790 PMC7887594

[B31] Cilluffo D. Barra V. Di Leonardo A. (2020). P14ARF: the absence that makes the difference. Genes (Basel) 11, 824. 10.3390/genes11070824 32698529 PMC7397060

[B32] Colle R. Lonardi S. Cachanado M. Overman M. J. Elez E. Fakih M. (2023). BRAF V600E/RAS mutations and Lynch syndrome in patients with MSI-H/dMMR metastatic colorectal cancer treated with immune checkpoint inhibitors. Oncologist 28, 771–779. 10.1093/oncolo/oyad082 37023721 PMC10485382

[B33] Corrales L. McWhirter S. M. Dubensky T. W. Gajewski T. F. (2016). The host STING pathway at the interface of cancer and immunity. J. Clin. Invest. 126, 2404–2411. 10.1172/JCI86892 27367184 PMC4922692

[B34] Cox C. Bignell G. Greenman C. Stabenau A. Warren W. Stephens P. (2005). A survey of homozygous deletions in human cancer genomes. Proc. Natl. Acad. Sci. U. S. A. 102, 4542–4547. 10.1073/pnas.0408593102 15761058 PMC555487

[B35] Czajkowski K. Herbet M. Murias M. Piątkowska-Chmiel I. (2025). Senolytics: charting a new course or enhancing existing anti-tumor therapies? Cell Oncol. (Dordr) 48, 351–371. 10.1007/s13402-024-01018-5 39633108 PMC11996976

[B36] Debniak T. Górski B. Huzarski T. Byrski T. Cybulski C. Mackiewicz A. (2005). A common variant of CDKN2A (p16) predisposes to breast cancer. J. Med. Genet. 42, 763–765. 10.1136/jmg.2005.031476 15879498 PMC1735931

[B37] Deng L. Liang H. Xu M. Yang X. Burnette B. Arina A. (2014). STING-dependent cytosolic DNA sensing promotes radiation-induced type I interferon-dependent antitumor immunity in immunogenic tumors. Immunity 41, 843–852. 10.1016/j.immuni.2014.10.019 25517616 PMC5155593

[B38] Dominguez G. Silva J. Garcia J. M. Silva J. M. Rodriguez R. Muñoz C. (2003). Prevalence of aberrant methylation of p14ARF over p16INK4a in some human primary tumors. Mutat. Res. 530, 9–17. 10.1016/s0027-5107(03)00133-7 14563526

[B39] Dong Y. Zheng M. Wang X. Yu C. Qin T. Shen X. (2023). High expression of CDKN2A is associated with poor prognosis in colorectal cancer and may guide PD-1-mediated immunotherapy. BMC Cancer 23, 1097. 10.1186/s12885-023-11603-w 37950153 PMC10638725

[B40] Dou J.-X. Zhang W.-D. Li W.-T. Li H.-L. Cai X.-S. Liu J. (2009). Expression of methylthioadenosine phosphorylase (MTAP) gene and demethylation of its promoter in human colorectal cancer. Ai Zheng 28, 390–394. 19622299

[B41] Drak Alsibai K. Vacher S. Meseure D. Nicolas A. Lae M. Schnitzler A. (2019). High positive correlations between ANRIL and p16-CDKN2A/p15-CDKN2B/p14-ARF gene cluster overexpression in multi-tumor types suggest deregulated activation of an ANRIL–ARF bidirectional promoter. Noncoding RNA 5, 44. 10.3390/ncrna5030044 31438464 PMC6789474

[B42] Eslinger C. Walden D. McGary A. Emiloju O. Ahn D. Sonbol M. B. (2025). Comparison of survival outcomes for patients with Lynch vs non-Lynch syndrome and microsatellite unstable colorectal cancer treated with immunotherapy. Cancer 131, e35756. 10.1002/cncr.35756 39932790

[B43] Esteller M. Tortola S. Toyota M. Capella G. Peinado M. A. Baylin S. B. (2000). Hypermethylation-associated inactivation of p14(ARF) is independent of p16(INK4a) methylation and p53 mutational status. Cancer Res. 60, 129–133. 10646864

[B44] Fan J. Li P. Fang Q. Yang Y. Zhang H. Du W. (2022). Heterotypic neutrophil-in-tumor structure: a novel pathological feature first discovered in the tissues of OPSCC. Front. Oncol. 12, 807597. 10.3389/fonc.2022.807597 36052249 PMC9425089

[B45] Farooq U. Notani D. (2022). Transcriptional regulation of INK4/ARF locus by cis and trans mechanisms. Front. Cell Dev. Biol. 10, 948351. 10.3389/fcell.2022.948351 36158211 PMC9500187

[B46] Fatemi N. Tierling S. Es H. A. Varkiani M. Mojarad E. N. Aghdaei H. A. (2022). DNA methylation biomarkers in colorectal cancer: clinical applications for precision medicine. Int. J. Cancer 151, 2068–2081. 10.1002/ijc.34186 35730647

[B47] Flatin B. T. B. Vedeld H. M. Pinto R. Langerud J. Lind G. E. Lothe R. A. (2021). Multiregional assessment of CIMP in primary colorectal cancers: phenotype concordance but marker variability. Int. J. Cancer 148, 1652–1657. 10.1002/ijc.33425 33284993 PMC7898891

[B48] Gallegos V. Rowdo F. M. White J. Kuo H.-H. Podaza E. Martin L. (2023). 344 the potential benefits of using senolytics in colorectal cancer treatment. J. Clin. Transl. Sci. 7, 102. 10.1017/cts.2023.388

[B49] Galluzzi L. Vanpouille-Box C. Bakhoum S. F. Demaria S. (2018). SnapShot: CGAS-STING signaling. Cell 173, 276–276.e1. 10.1016/j.cell.2018.03.015 29570996

[B50] Gao Z. Li W. Mao X. Huang T. Wang H. Li Y. (2021). Single-nucleotide methylation specifically represses type I interferon in antiviral innate immunity. J. Exp. Med. 218, e20201798. 10.1084/jem.20201798 33616624 PMC7903198

[B51] Gil J. Peters G. (2006). Regulation of the INK4b-ARF-INK4a tumour suppressor locus: all for one or one for all. Nat. Rev. Mol. Cell Biol. 7, 667–677. 10.1038/nrm1987 16921403

[B52] Gorgoulis V. Adams P. D. Alimonti A. Bennett D. C. Bischof O. Bishop C. (2019). Cellular senescence: defining a path forward. Cell 179, 813–827. 10.1016/j.cell.2019.10.005 31675495

[B53] Gounder M. Johnson M. Heist R. S. Shapiro G. I. Postel-Vinay S. Wilson F. H. (2025). MAT2A inhibitor AG-270/S095033 in patients with advanced malignancies: a phase I trial. Nat. Commun. 16, 423. 10.1038/s41467-024-55316-5 39762248 PMC11704051

[B54] Grasmann G. Smolle E. Olschewski H. Leithner K. (2019). Gluconeogenesis in cancer cells – repurposing of a starvation-induced metabolic pathway? Biochim. Biophys. Acta Rev. Cancer 1872, 24–36. 10.1016/j.bbcan.2019.05.006 31152822 PMC6894939

[B55] Graziano F. Ruzzo A. Giacomini E. Ricciardi T. Aprile G. Loupakis F. (2017). Glycolysis gene expression analysis and selective metabolic advantage in the clinical progression of colorectal cancer. Pharmacogenomics J. 17, 258–264. 10.1038/tpj.2016.13 26927284

[B56] Greco L. Rubbino F. Dal Buono A. Laghi L. (2023). Microsatellite instability and immune response: from microenvironment features to therapeutic Actionability—Lessons from colorectal cancer. Genes (Basel) 14, 1169. 10.3390/genes14061169 37372349 PMC10298406

[B57] Guillon J. Petit C. Moreau M. Toutain B. Henry C. Roché H. (2019). Regulation of senescence escape by TSP1 and CD47 following chemotherapy treatment. Cell Death Dis. 10, 199. 10.1038/s41419-019-1406-7 30814491 PMC6393582

[B58] Guo J. K. Blanco M. R. Walkup W. G. Bonesteele G. Urbinati C. R. Banerjee A. K. (2024). Denaturing purifications demonstrate that PRC2 and other widely-reported chromatin proteins do not appear to bind directly to RNA *in vivo* . Mol. Cell 84, 1271–1289.e12. 10.1016/j.molcel.2024.01.026 38387462 PMC10997485

[B59] Gutierrez W. R. Scherer A. Rytlewski J. D. Laverty E. A. Sheehan A. P. McGivney G. R. (2022). Augmenting chemotherapy with low-dose decitabine through an immune-independent mechanism. JCI Insight 7, e159419. 10.1172/jci.insight.159419 36227698 PMC9746804

[B60] Haferkamp S. Tran S. L. Becker T. M. Scurr L. L. Kefford R. F. Rizos H. (2009). The relative contributions of the p53 and pRb pathways in oncogene-induced melanocyte senescence. Aging (Albany NY) 1, 542–556. 10.18632/aging.100051 20157537 PMC2806033

[B61] Han G. Yang G. Hao D. Lu Y. Thein K. Simpson B. S. (2021). 9p21 loss confers a cold tumor immune microenvironment and primary resistance to immune checkpoint therapy. Nat. Commun. 12, 5606. 10.1038/s41467-021-25894-9 34556668 PMC8460828

[B62] Hariharan R. Jenkins M. (2020). Utility of the methylated SEPT9 test for the early detection of colorectal cancer: a systematic review and meta-analysis of diagnostic test accuracy. BMJ Open Gastroenterol. 7, e000355. 10.1136/bmjgast-2019-000355 32128229 PMC7039590

[B63] Harismendy O. Notani D. Song X. Rahim N. G. Tanasa B. Heintzman N. (2011). 9p21 DNA variants associated with coronary artery disease impair IFNγ signaling response. Nature 470, 264–268. 10.1038/nature09753 21307941 PMC3079517

[B64] Harmonizome 3.0: Focad (2025). Available online at: https://maayanlab.cloud/Harmonizome/gene/FOCAD (Accessed August 18, 2025).

[B65] Hayashi Y. Raimondi S. C. Look A. T. (1991). “Molecular analysis of chromosomal abnormalities in Childhood acute lymphoblastic leukemia,” in Childhood leukemia: present problems and future prospects. Editors Kobayashi N. Akera T. Mizutani S. (Boston, MA: Springer US), 59–68. 10.1007/978-1-4615-3898-1_6

[B66] He X. Zhong X. Fang Y. Hu Z. Chen Z. Wang Y. (2023). AF9 sustains glycolysis in colorectal cancer *via* H3K9ac‐mediated PCK2 and FBP1 transcription. Clin. Trans. Med. 13, e1352. 10.1002/ctm2.1352 37565737 PMC10413954

[B67] Healy E. Zhang Q. Gail E. H. Agius S. C. Sun G. Bullen M. (2024). The apparent loss of PRC2 chromatin occupancy as an artifact of RNA depletion. Cell Rep. 43, 113858. 10.1016/j.celrep.2024.113858 38416645

[B68] Helwick C. (2024). First-line nivolumab plus ipilimumab shows benefit in metastatic colorectal cancer subset. Available online at: https://ascopost.com/news/january-2024/first-line-nivolumab-plus-ipilimumab-shows-benefit-in-metastatic-colorectal-cancer-subset/(Accessed January 3, 2026).

[B69] Hinoue T. Weisenberger D. J. Lange C. P. E. Shen H. Byun H.-M. Van Den Berg D. (2012). Genome-scale analysis of aberrant DNA methylation in colorectal cancer. Genome Res. 22, 271–282. 10.1101/gr.117523.110 21659424 PMC3266034

[B70] Hirosue A. Ishihara K. Tokunaga K. Watanabe T. Saitoh N. Nakamoto M. (2012). Quantitative assessment of higher-order chromatin structure of the INK4/ARF locus in human senescent cells. Aging Cell 11, 553–556. 10.1111/j.1474-9726.2012.00809.x 22340434

[B71] Hong J. Lee J. H. Zhang Z. Wu Y. Yang M. Liao Y. (2022). PRC2-mediated epigenetic suppression of type I IFN-STAT2 signaling impairs antitumor immunity in luminal breast cancer. Cancer Research 82, 4624–4640. 10.1158/0008-5472.CAN-22-0736 36222718 PMC9772098

[B72] Horn I. P. Marks D. L. Koenig A. N. Hogenson T. L. Almada L. L. Goldstein L. E. (2021). A rare germline CDKN2A variant (47T>G; p16-L16R) predisposes carriers to pancreatic cancer by reducing cell cycle inhibition. J. Biol. Chem. 296, 100634. 10.1016/j.jbc.2021.100634 33823155 PMC8121974

[B73] Hoy S. M. (2020). Tazemetostat: first approval. Drugs 80, 513–521. 10.1007/s40265-020-01288-x 32166598

[B74] Huang J. Yin Q. Wang Y. Zhou X. Guo Y. Tang Y. (2024a). EZH2 inhibition enhances PD‐L1 protein stability through USP22‐Mediated deubiquitination in colorectal cancer. Adv. Sci. (Weinh) 11, 2308045. 10.1002/advs.202308045 38520088 PMC11187912

[B75] Huang K. C.-Y. Ke T.-W. Lai C.-Y. Hong W.-Z. Chang H.-Y. Lee C.-Y. (2024b). Inhibition of DNMTs increases neoantigen-reactive T-cell toxicity against microsatellite-stable colorectal cancer in combination with radiotherapy. Biomed. Pharmacother. 177, 116958. 10.1016/j.biopha.2024.116958 38917760

[B76] Huang C. Gao Y. Chen J. Hong J. H. Jiang Y. Chai K. X. Y. (2025). Priming with DNMT inhibitors potentiates PD-1 immunotherapy by triggering viral mimicry in relapsed/refractory NK/T-cell lymphoma. Cancer Discov. 15, 2450–2467. 10.1158/2159-8290.CD-25-0587 41088524 PMC12670078

[B77] Hyung J. Cho E. J. Kim J. Kim J. H. Kim J. E. Hong Y. S. (2022). Histopathologic and molecular biomarkers of PD-1/PD-L1 inhibitor treatment response among patients with microsatellite instability–high Colon cancer. Cancer Res. Treat. 54, 1175–1190. 10.4143/crt.2021.1133 35038827 PMC9582482

[B78] Ishiguro A. Takahata T. Saito M. Yoshiya G. Tamura Y. Sasaki M. (2006). Influence of methylated p15 and p16 genes on clinicopathological features in colorectal cancer. J. Gastroenterol. Hepatol. 21, 1334–1339. 10.1111/j.1440-1746.2006.04137.x 16872319

[B79] Islam Z. Saravanan B. Walavalkar K. Farooq U. Singh A. K. Radhakrishnan S. (2023). Active enhancers strengthen insulation by RNA-Mediated CTCF binding at chromatin domain boundaries. Genome Res. 33, 1–17. 10.1101/gr.276643.122 36650052 PMC9977152

[B80] Ivashkiv L. B. Donlin L. T. (2014). Regulation of type I interferon responses. Nat. Rev. Immunol. 14, 36–49. 10.1038/nri3581 24362405 PMC4084561

[B81] Iwasaki A. (2025). cGAS detects cytosolic DNA. Available online at: https://app.biorender.com/biorender-templates/details/t-6298c05cdf2c4758fec28b0a-cgas-detects-cytosolic-dna (Accessed August 18, 2025).

[B82] Joo J. E. Mahmood K. Walker R. Georgeson P. Candiloro I. Clendenning M. (2023). Identifying primary and secondary MLH1 epimutation carriers displaying low-level constitutional MLH1 methylation using droplet digital PCR and genome-wide DNA methylation profiling of colorectal cancers. Clin. Epigenetics 15, 95. 10.1186/s13148-023-01511-y 37270516 PMC10239107

[B83] Jung G. Hernández-Illán E. Moreira L. Balaguer F. Goel A. (2020). Epigenetics of colorectal cancer: biomarker and therapeutic potential. Nat. Rev. Gastroenterol. Hepatol. 17, 111–130. 10.1038/s41575-019-0230-y 31900466 PMC7228650

[B84] Kabirova E. Nurislamov A. Shadskiy A. Smirnov A. Popov A. Salnikov P. (2023). Function and evolution of the loop extrusion machinery in animals. Int. J. Mol. Sci. 24, 5017. 10.3390/ijms24055017 36902449 PMC10003631

[B85] Karam R. A. Zidan H. E. Abd Elrahman T. M. Badr S. A. Amer S. A. (2019). Study of p16 promoter methylation in Egyptian colorectal cancer patients. J. Cell Biochem. 120, 8581–8587. 10.1002/jcb.28146 30485512

[B86] Katayama T. Nakanishi K. Nishihara H. Kamiyama N. Nakagawa T. Kamiyama T. (2007). Type I interferon prolongs cell cycle progression *via* p21WAF1/CIP1 induction in human colon cancer cells. Int. J. Oncol. 31, 613–620. 10.3892/ijo.31.3.613 17671689

[B87] Katlinskaya Y. V. Carbone C. J. Yu Q. Fuchs S. Y. (2015). Type 1 interferons contribute to the clearance of senescent cell. Cancer Biol. Ther. 16, 1214–1219. 10.1080/15384047.2015.1056419 26046815 PMC4622626

[B88] Katlinskaya Y. V. Katlinski K. V. Yu Q. Ortiz A. Beiting D. P. Brice A. (2016). Suppression of type I interferon signaling overcomes oncogene-induced senescence and mediates melanoma development and progression. Cell Rep. 15, 171–180. 10.1016/j.celrep.2016.03.006 27052162 PMC4826807

[B89] Kedrin D. Gala M. K. (2015). Genetics of the serrated pathway to colorectal cancer. Clin. Transl. Gastroenterol. 6, e84. 10.1038/ctg.2015.12 25856207 PMC4459531

[B90] Khosla S. (2024). Targeting cellular senescence with senolytics to improve skeletal health in older humans: a phase 2, Single-Center, 20-week, Open-Label, randomized controlled trial. clinicaltrials.gov. Available online at: https://clinicaltrials.gov/study/NCT04313634 (Accessed August 18, 2025).

[B91] Kibriya M. G. Jasmine F. Khamkevych Y. Raza M. Kamal M. Bissonnette M. (2024). Association of Microsatellite instability and gene expression profile in colorectal carcinoma and potential implications for therapy. Med. Kaunas. 60, 348. 10.3390/medicina60030348 38541076 PMC10972457

[B209] Kim D. J. (2025). The role of the DNA methyltransferase family and the therapeutic potential of DNMT inhibitors in tumor treatment. Curr. Oncol. 32, 88. 10.3390/curroncol32020088 39996888 PMC11854558

[B92] Kim J. H. Kang G. H. (2014). Molecular and prognostic heterogeneity of microsatellite-unstable colorectal cancer. World J. Gastroenterol. 20, 4230–4243. 10.3748/wjg.v20.i15.4230 24764661 PMC3989959

[B93] Kim W. Y. Sharpless N. E. (2006). The regulation of INK4/ARF in cancer and aging. Cell 127, 265–275. 10.1016/j.cell.2006.10.003 17055429

[B94] Kim J. H. Kim K.-J. Rhee Y.-Y. Oh S. Cho N.-Y. Lee H. S. (2014). Expression status of wild-type HSP110 correlates with HSP110 T17 deletion size and patient prognosis in microsatellite-unstable colorectal cancer. Mod. Pathol. 27, 443–453. 10.1038/modpathol.2013.160 24030751

[B95] Kim Y. H. Choi Y. W. Lee J. Soh E. Y. Kim J.-H. Park T. J. (2017). Senescent tumor cells lead the collective invasion in thyroid cancer. Nat. Commun. 8, 15208. 10.1038/ncomms15208 28489070 PMC5436223

[B96] Kim J. H. Hong J. Lee J. A. Jung M. Choi E. Cho N.-Y. (2024). Immune microenvironmental heterogeneity according to tumor DNA methylation phenotypes in microsatellite instability-high colorectal cancers. Cancer Immunol. Immunother. 73, 215. 10.1007/s00262-024-03805-3 39235590 PMC11377388

[B97] Koi M. Tseng-Rogenski S. S. Carethers J. M. (2018). Inflammation-associated microsatellite alterations: mechanisms and significance in the prognosis of patients with colorectal cancer. World J. Gastrointest. Oncol. 10, 1–14. 10.4251/wjgo.v10.i1.1 29375743 PMC5767788

[B98] Kotake Y. Nakagawa T. Kitagawa K. Suzuki S. Liu N. Kitagawa M. (2011). Long non-coding RNA ANRIL is required for the PRC2 recruitment to and silencing of p15INK4B tumor suppressor gene. Oncogene 30, 1956–1962. 10.1038/onc.2010.568 21151178 PMC3230933

[B99] Kuang C. Park Y. Augustin R. C. Lin Y. Hartman D. J. Seigh L. (2022). Pembrolizumab plus azacitidine in patients with chemotherapy refractory metastatic colorectal cancer: a single-arm phase 2 trial and correlative biomarker analysis. Clin. Epigenetics 14, 3. 10.1186/s13148-021-01226-y 34991708 PMC8740438

[B100] Kuismanen S. A. Holmberg M. T. Salovaara R. de la Chapelle A. Peltomäki P. (2000). Genetic and epigenetic modification of MLH1 accounts for a major share of microsatellite-unstable colorectal cancers. Am. J. Pathol. 156, 1773–1779. 10.1016/S0002-9440(10)65048-1 10793088 PMC1876911

[B101] Lam Y. Gu J. Yin P. (2025). Cellular senescence in cancer: unveiling dual roles, tumor microenvironment dynamics and therapeutic innovations. Oncol. Lett. 30, 1–16. 10.3892/ol.2025.15338 PMC12557303 41158311

[B102] Le Duff M. Gouju J. Jonchère B. Guillon J. Toutain B. Boissard A. (2018). Regulation of senescence escape by the cdk4–EZH2–AP2M1 pathway in response to chemotherapy. Cell Death Dis. 9, 199. 10.1038/s41419-017-0209-y 29415991 PMC5833455

[B103] Lee H. Saw A. Morris V. Singh A. Napolitano S. Sorokin A. (2024). Abstract 3241: elevated H3K27 trimethylation mediates adaptation to DNA demethylation in BRAFV600E-mutated colorectal cancer. Cancer Res. 84, 3241. 10.1158/1538-7445.AM2024-3241

[B104] Leerhoff S. Raem A. Kolbe E.-W. Schulz L. Borchers K. Köhler T. (2023). Methylated Septin9 identified patients with colorectal carcinoma and showed higher sensitivity than conventional biomarkers in detecting tumor. Cancer Treat. Res. Commun. 36, 100748. 10.1016/j.ctarc.2023.100748 37541105

[B105] Lenz H.-J. Lonardi S. Elez E. Van Cutsem E. Jensen L. H. Bennouna J. (2024). Nivolumab (NIVO) plus ipilimumab (IPI) vs chemotherapy (chemo) as first-line (1L) treatment for microsatellite instability-high/mismatch repair-deficient (MSI-H/dMMR) metastatic colorectal cancer (mCRC): expanded efficacy analysis from CheckMate 8HW. J. Clin. Oncol. 42, 3503. 10.1200/JCO.2024.42.16_suppl.3503

[B106] Li W.-Q. Pfeiffer R. M. Hyland P. L. Shi J. Gu F. Wang Z. (2014a). Genetic polymorphisms in the 9p21 region associated with risk of multiple cancers. Carcinogenesis 35, 2698–2705. 10.1093/carcin/bgu203 25239644 PMC4247519

[B107] Li Y. Wen H. Xi Y. Tanaka K. Wang H. Peng D. (2014b). AF9 YEATS domain links histone acetylation to DOT1L-Mediated H3K79 methylation. Cell 159, 558–571. 10.1016/j.cell.2014.09.049 25417107 PMC4344132

[B108] Li Y. Sabari B. R. Panchenko T. Wen H. Zhao D. Guan H. (2016). Molecular coupling of histone crotonylation and active transcription by AF9 YEATS domain. Mol. Cell 62, 181–193. 10.1016/j.molcel.2016.03.028 27105114 PMC4841940

[B109] Li C. Zhao X. He Y. Li Z. Qian J. Zhang L. (2022a). The functional role of inherited CDKN2A variants in childhood acute lymphoblastic leukemia. Pharmacogenet Genomics 32, 43–50. 10.1097/FPC.0000000000000451 34369425 PMC8694244

[B110] Li Z. Qiao J. Ma W. Zhou J. Gu L. Deng D. (2022b). P14AS upregulates gene expression in the CDKN2A/2B locus through competitive binding to PcG protein CBX7. Front. Cell Dev. Biol. 10, 993525. 10.3389/fcell.2022.993525 36176277 PMC9513069

[B111] Li Q. Liu X. Wen J. Chen X. Xie B. Zhao Y. (2023). Enhancer RNAs: mechanisms in transcriptional regulation and functions in diseases. Cell Commun. Signal. 21, 191. 10.1186/s12964-023-01206-0 37537618 PMC10398997

[B112] Linnekamp J. F. Kandimalla R. Fessler E. de Jong J. H. Rodermond H. M. van Bochove G. G. W. (2021). Pre-Operative decitabine in Colon cancer patients: analyses on WNT target methylation and expression. Cancers (Basel) 13, 2357. 10.3390/cancers13102357 34068407 PMC8153633

[B113] Liu P. Wu D. Duan J. Xiao H. Zhou Y. Zhao L. (2020). NRF2 regulates the sensitivity of human NSCLC cells to cystine deprivation-induced ferroptosis *via* FOCAD-FAK signaling pathway. Redox Biol. 37, 101702. 10.1016/j.redox.2020.101702 32898818 PMC7486457

[B114] López J. Llop-Hernández À. Verdura S. Serrano-Hervás E. Martinez-Balibrea E. Bosch-Barrera J. (2025). Mitochondrial priming and response to BH3 mimetics in “one-two punch” senogenic-senolytic strategies. Cell Death Discov. 11, 91. 10.1038/s41420-025-02379-y 40055336 PMC11889205

[B115] Lu S. Han L. Hu X. Sun T. Xu D. Li Y. (2021). N6-methyladenosine reader IMP2 stabilizes the ZFAS1/OLA1 axis and activates the Warburg effect: implication in colorectal cancer. J. Hematol. Oncol. 14, 188. 10.1186/s13045-021-01204-0 34743750 PMC8574039

[B116] Lugano G. (2025). Intracellular pathway mediated by interferons in psoriasis. Available online at: https://app.biorender.com/biorender-templates/details/t-6664a37f9c9ba416976ca415-intracellular-pathway-mediated-by-interferons-in-psoriasis (Accessed August 18, 2025).

[B117] Luo Y. Liang G. Zhang Q. Luo B. (2024). The role of cGAS-STING signaling pathway in colorectal cancer immunotherapy: mechanism and progress. Int. Immunopharmacol. 143, 113447. 10.1016/j.intimp.2024.113447 39515043

[B210] Mansfield L. Ramponi V. Gupta K. Stevenson T. Mathew A. B. Barinda A. J. (2024). Emerging insights in senescence: pathways from preclinical models to therapeutic innovations. NPJ Aging 10, 53. 10.1038/s41514-024-00181-1 39578455 PMC11584693

[B118] Mauri G. Patelli G. Roazzi L. Valtorta E. Amatu A. Marrapese G. (2024). Clinicopathological characterisation of MTAP alterations in gastrointestinal cancers. J. Clin. Pathol. 78, e209341-201. 10.1136/jcp-2023-209341 38350716 PMC11874331

[B119] Mayo Clinic (2025). Pilot Study of the mechanistic feedback from CNS tumors with latent residual disease to guide individualized therapies. clinicaltrials.gov. Available online at: https://clinicaltrials.gov/study/NCT07025226 (Accessed August 18, 2025).

[B120] McNab F. Mayer-Barber K. Sher A. Wack A. O’Garra A. (2015). Type I interferons in infectious disease. Nat. Rev. Immunol. 15, 87–103. 10.1038/nri3787 25614319 PMC7162685

[B121] McRonald F. E. Pethick J. Santaniello F. Shand B. Tyson A. Tulloch O. (2024). Identification of people with Lynch syndrome from those presenting with colorectal cancer in England: baseline analysis of the diagnostic pathway. Eur. J. Hum. Genet. 32, 529–538. 10.1038/s41431-024-01550-w 38355963 PMC11061113

[B122] Mender I. Zhang A. Ren Z. Han C. Deng Y. Siteni S. (2020). Telomere stress potentiates STING-dependent anti-tumor immunity. Cancer Cell 38, 400–411.e6. 10.1016/j.ccell.2020.05.020 32619407 PMC7494563

[B123] Mödl B. Moritsch S. Zwolanek D. Eferl R. (2023). Type I and II interferon signaling in colorectal cancer liver metastasis. Cytokine 161, 156075. 10.1016/j.cyto.2022.156075 36323190

[B124] Moreno-Ortiz J. M. Jiménez-García J. Gutiérrez-Angulo M. Ayala-Madrigal M. de la L. González-Mercado A. González-Villaseñor C. O. (2021). High frequency of MLH1 promoter methylation mediated by gender and age in colorectal tumors from Mexican patients. Gac. Med. Mex. 157, 618–623. 10.24875/GMM.M21000626 35108246

[B125] Morgan E. Arnold M. Gini A. Lorenzoni V. Cabasag C. J. Laversanne M. (2023). Global burden of colorectal cancer in 2020 and 2040: incidence and mortality estimates from GLOBOCAN. Gut 72, 338–344. 10.1136/gutjnl-2022-327736 36604116

[B126] Muniz L. Lazorthes S. Delmas M. Ouvrard J. Aguirrebengoa M. Trouche D. (2020). Circular ANRIL isoforms switch from repressors to activators of p15/CDKN2B expression during RAF1 oncogene-induced senescence. RNA Biol. 18, 404–420. 10.1080/15476286.2020.1812910 32862732 PMC7951966

[B127] Musella M. Galassi C. Manduca N. Sistigu A. (2021). The yin and Yang of type I IFNs in cancer promotion and immune activation. Biol. (Basel) 10, 856. 10.3390/biology10090856 34571733 PMC8467547

[B128] Naemura M. Tsunoda T. Inoue Y. Okamoto H. Shirasawa S. Kotake Y. (2016). ANRIL regulates the proliferation of human colorectal cancer cells in both two- and three-dimensional culture. Mol. Cell Biochem. 412, 141–146. 10.1007/s11010-015-2618-5 26708220

[B129] National Cancer Institute (NCI) (2014). A phase II Study of dasatinib (NSC 732517) in previously-treated patients with metastatic colorectal cancer. clinicaltrials.gov. Available online at: https://clinicaltrials.gov/study/NCT00504153 (Accessed August 19, 2025).

[B130] National Cancer Institute (NCI) (2025). An open label, two-part, phase Ib/II Study to investigate the safety, pharmacokinetics, pharmacodynamics, and clinical activity of the MEK inhibitor trametinib and the BCL2-Family inhibitor navitoclax (ABT-263) in combination in subjects with KRAS or NRAS mutation-positive advanced solid tumors. clinicaltrials.gov. Available online at: https://clinicaltrials.gov/study/NCT02079740 (Accessed August 19, 2025).

[B131] Ngoi N. Y. L. Tang T.-Y. Gaspar C. F. Pavlick D. C. Buchold G. M. Scholefield E. L. (2024). Methylthioadenosine phosphorylase genomic loss in advanced gastrointestinal cancers. Oncologist 29, 493–503. 10.1093/oncolo/oyae011 38330461 PMC11144995

[B132] Nguyen L. H. Goel A. Chung D. C. (2020). Pathways of colorectal carcinogenesis. Gastroenterology 158, 291–302. 10.1053/j.gastro.2019.08.059 31622622 PMC6981255

[B133] Nian J. Sun X. Ming S. Yan C. Ma Y. Feng Y. (2017). Diagnostic accuracy of methylated SEPT9 for blood-based colorectal cancer detection: a systematic review and meta-analysis. Clin. Transl. Gastroenterol. 8, e216. 10.1038/ctg.2016.66 28102859 PMC5288600

[B134] Nicolas A. M. Pesic M. Engel E. Ziegler P. K. Diefenhardt M. Kennel K. B. (2022). Inflammatory fibroblasts mediate resistance to neoadjuvant therapy in rectal cancer. Cancer Cell 40, 168–184.e13. 10.1016/j.ccell.2022.01.004 35120600

[B135] Nieminen T. T. Shoman S. Eissa S. Peltomäki P. Abdel-Rahman W. M. (2012). Distinct genetic and epigenetic signatures of colorectal cancers according to ethnic origin. Cancer Epidemiol. Biomarkers Prev. 21, 202–211. 10.1158/1055-9965.EPI-11-0662 22028395

[B136] Nilsson T. K. Löf-Öhlin Z. M. Sun X.-F. (2012). DNA methylation of the p14ARF, RASSF1A and APC1A genes as an independent prognostic factor in colorectal cancer patients. Int. J. Oncol. 42, 127–133. 10.3892/ijo.2012.1682 23128528 PMC3583697

[B137] Nosho K. Irahara N. Shima K. Kure S. Kirkner G. J. Schernhammer E. S. (2008). Comprehensive biostatistical analysis of CpG Island methylator phenotype in colorectal cancer using a large population-based sample. PLoS One 3, e3698. 10.1371/journal.pone.0003698 19002263 PMC2579485

[B138] Ogino S. Kawasaki T. Kirkner G. J. Kraft P. Loda M. Fuchs C. S. (2007). Evaluation of markers for CpG Island Methylator Phenotype (CIMP) in colorectal cancer by a large population-based sample. J. Mol. Diagn. 9, 305–314. 10.2353/jmoldx.2007.060170 17591929 PMC1899428

[B139] Oh C. K. Cho Y.-S. (2024). Pathogenesis and biomarkers of colorectal cancer by epigenetic alteration. Intest. Res. 22, 131–151. 10.5217/ir.2023.00115 38295766 PMC11079515

[B140] Orleni M. Beumer J. H. (2024). Pharmacology and pharmacokinetics of tazemetostat. Cancer Chemother. Pharmacol. 93, 509–517. 10.1007/s00280-024-04658-4 38520556 PMC11559081

[B141] Overman M. J. Morris V. Moinova H. Manyam G. Ensor J. Lee M. S. (2016). Phase I/II study of azacitidine and capecitabine/oxaliplatin (CAPOX) in refractory CIMP-high metastatic colorectal cancer: evaluation of circulating methylated vimentin. Oncotarget 7, 67495–67506. 10.18632/oncotarget.11317 27542211 PMC5341892

[B142] Ozenne P. Eymin B. Brambilla E. Gazzeri S. (2010). The ARF tumor suppressor: structure, functions and status in cancer. Int. J. Cancer 127, 2239–2247. 10.1002/ijc.25511 20549699

[B143] Palmieri L. J. Cousin S. Spalato M. Guégan J. P. Bessede A. Italiano A. (2023). Targeting EZH2 to overcome the resistance to immunotherapy in microsatellite stable colorectal cancer: results from the CAIRE study. J. Clin. Oncol. 41, 3599. 10.1200/JCO.2023.41.16_suppl.3599

[B144] Patel P. L. Suram A. Mirani N. Bischof O. Herbig U. (2016). Derepression of hTERT gene expression promotes escape from oncogene-induced cellular senescence. Proc. Natl. Acad. Sci. U. S. A. 113, E5024–E5033. 10.1073/pnas.1602379113 27503890 PMC5003242

[B145] Patro C. P. K. Biswas N. Pingle S. C. Lin F. Anekoji M. Jones L. D. (2022). MTAP loss: a possible therapeutic approach for glioblastoma. J. Transl. Med. 20, 620. 10.1186/s12967-022-03823-8 36572880 PMC9791736

[B146] Platanitis E. Gruener S. Ravi Sundar Jose Geetha A. Boccuni L. Vogt A. Novatchkova M. (2022). Interferons reshape the 3D conformation and accessibility of macrophage chromatin. iScience 25, 103840. 10.1016/j.isci.2022.103840 35243225 PMC8857492

[B147] Pukhalskaia T. V. Yurakova T. R. Bogdanova D. A. Demidov O. N. (2024). Tumor-Associated senescent macrophages, their markers, and their role in tumor microenvironment. Biochem. (Mosc) 89, 839–852. 10.1134/S0006297924050055 38880645

[B148] Rahman M. Olson I. Mansour M. Carlstrom L. P. Sutiwisesak R. Saber R. (2022). Selective vulnerability of senescent glioblastoma cells to BCL-XL inhibition. Mol. Cancer Res. 20, 938–948. 10.1158/1541-7786.MCR-21-0029 35191501 PMC9196639

[B149] Rao S. S. P. Huntley M. H. Durand N. C. Stamenova E. K. Bochkov I. D. Robinson J. T. (2014). A three-dimensional map of the human genome at kilobase resolution reveals principles of chromatin looping. Cell 159, 1665–1680. 10.1016/j.cell.2014.11.021 25497547 PMC5635824

[B150] Rawson J. B. Bapat B. (2012). Epigenetic biomarkers in colorectal cancer diagnostics. Expert Rev. Mol. Diagn. 12, 499–509. 10.1586/erm.12.39 22702366

[B151] Reddel R. R. (2010). Senescence: an antiviral defense that is tumor suppressive? Carcinogenesis 31, 19–26. 10.1093/carcin/bgp274 19887513

[B152] Reyila A. Gao X. Yu J. Nie Y. (2025). Insight into the role of DNA methylation in prognosis and treatment response prediction of gastrointestinal cancers. Epigenomics 17, 475–488. 10.1080/17501911.2025.2476380 40084815 PMC12026041

[B153] Rico-Méndez M. A. Trujillo-Rojas M. A. Ayala-Madrigal M. de la L. Hernández-Sandoval J. A. González-Mercado A. Gutiérrez-Angulo M. (2025). MLH1 methylation status and microsatellite instability in patients with colorectal cancer. Genes (Basel) 16, 182. 10.3390/genes16020182 40004511 PMC11854980

[B154] Roulois D. Loo Yau H. Singhania R. Wang Y. Danesh A. Shen S. Y. (2015). DNA-Demethylating agents target colorectal cancer cells by inducing viral mimicry by endogenous transcripts. Cell 162, 961–973. 10.1016/j.cell.2015.07.056 26317465 PMC4843502

[B155] Russo M. Moccia S. Luongo D. Russo G. L. (2023). Senolytic flavonoids enhance type-I and Type-II cell death in human radioresistant Colon cancer cells through AMPK/MAPK pathway. Cancers (Basel) 15, 2660. 10.3390/cancers15092660 37174126 PMC10177236

[B156] Ryan E. L. Hollingworth R. Grand R. J. (2016). Activation of the DNA damage response by RNA viruses. Biomolecules 6, 2. 10.3390/biom6010002 26751489 PMC4808796

[B157] Sahin I. H. Chakrabarti S. Hsieh R. W. Bruno T. C. Selfridge J. E. Gorantla V. (2024). Combining low-dose regorafenib with pembrolizumab for patients with MSI-H colorectal cancer: REGPEM-CRC-01. J. Clin. Oncol. 42, TPS238. 10.1200/JCO.2024.42.3_suppl.TPS238

[B158] Salar A. Vuković Đerfi K. Pačić A. Škrtić A. Cacev T. Kapitanović S. (2024). Association of functional polymorphisms in MSH3 and IL-6 pathway genes with different types of microsatellite instability in sporadic colorectal cancer. Cancers 16, 2916. 10.3390/cancers16162916 39199686 PMC11353200

[B159] Samson N. Ablasser A. (2022). The cGAS–STING pathway and cancer. Nat. Cancer 3, 1452–1463. 10.1038/s43018-022-00468-w 36510011

[B160] Sano T. Oyama T. Kashiwabara K. Fukuda T. Nakajima T. (1998). Expression status of p16 protein is associated with human papillomavirus oncogenic potential in cervical and genital lesions. Am. J. Pathol. 153, 1741–1748. 10.1016/S0002-9440(10)65689-1 9846965 PMC1866324

[B161] Schmitt C. A. Wang B. Demaria M. (2022). Senescence and cancer — role and therapeutic opportunities. Nat. Rev. Clin. Oncol. 19, 619–636. 10.1038/s41571-022-00668-4 36045302 PMC9428886

[B162] Seppälä T. T. Böhm J. P. Friman M. Lahtinen L. Väyrynen V. M. J. Liipo T. K. E. (2015). Combination of microsatellite instability and BRAF mutation status for subtyping colorectal cancer. Br. J. Cancer 112, 1966–1975. 10.1038/bjc.2015.160 25973534 PMC4580394

[B163] Shao Z. (2024). A pilot Study to explore the efficacy and safety of dasatinib combined with Quercetin to reverse chemotherapy resistance in triple negative breast cancer. clinicaltrials.gov. Available online at: https://clinicaltrials.gov/study/NCT06355037 (Accessed August 18, 2025).

[B164] Shi W.-K. Li Y.-H. Bai X.-S. Lin G.-L. (2022). The cell cycle-associated protein CDKN2A May promotes colorectal cancer cell metastasis by inducing epithelial-mesenchymal transition. Front. Oncol. 12, 834235. 10.3389/fonc.2022.834235 35311137 PMC8929760

[B165] Shima K. Nosho K. Baba Y. Cantor M. Meyerhardt J. A. Giovannucci E. L. (2011). Prognostic significance of CDKN2A (p16) promoter methylation and loss of expression in 902 colorectal cancers: Cohort Study and literature review. Int. J. Cancer 128, 1080–1094. 10.1002/ijc.25432 20473920 PMC2958235

[B166] Silva T. D. Vidigal V. M. Felipe A. V. De Lima J. M. Neto R. A. Saad S. S. (2013). DNA methylation as an epigenetic biomarker in colorectal cancer. Oncol. Lett. 6, 1687–1692. 10.3892/ol.2013.1606 24260063 PMC3834199

[B167] Sistigu A. Yamazaki T. Vacchelli E. Chaba K. Enot D. P. Adam J. (2014). Cancer cell-autonomous contribution of type I interferon signaling to the efficacy of chemotherapy. Nat. Med. 20, 1301–1309. 10.1038/nm.3708 25344738

[B168] Song P. Yang F. (2025). Protein arginine methyltransferase 5 as a novel therapeutic target in solid tumors. Genes Dis. 13, 101796. 10.1016/j.gendis.2025.101796 41078957 PMC12513006

[B169] Spiliopoulou P. Yang S. Y. C. Bruce J. P. Wang B. X. Berman H. K. Pugh T. J. (2022). All is not lost: learning from 9p21 loss in cancer. Trends Immunol. 43, 379–390. 10.1016/j.it.2022.03.003 35379580

[B170] St. Jude Children’s Research Hospital (2025). SEN-SURVIVORS: an open-label intervention trial to reduce senescence and improve frailty in adult survivors of childhood cancer. clinicaltrials.gov. Available online at: https://clinicaltrials.gov/study/NCT04733534 (Accessed August 18, 2025).

[B171] Straining R. Eighmy W. (2022). Tazemetostat: EZH2 inhibitor. J. Adv. Pract. Oncol. 13, 158–163. 10.6004/jadpro.2022.13.2.7 35369397 PMC8955562

[B172] Su T. Zhang Y. Valerie K. Wang X.-Y. Lin S. Zhu G. (2019). STING activation in cancer immunotherapy. Theranostics 9, 7759–7771. 10.7150/thno.37574 31695799 PMC6831454

[B173] Subramaniam B. Chong W. C. Babaei A. Bornhorst M. Zhang C. Packer R. (2025). MTAP-Null tumors: a comprehensive review on synthetic vulnerabilities and therapeutic strategies. Cells 14, 1964. 10.3390/cells14241964 41439984 PMC12732168

[B174] Sun Y. Liu Y. Jiang L. Zhong C. (2025). m5C methylation modification may be an accomplice in colorectal cancer escaping from anti-tumor effects of innate immunity-type I/III interferon. Front. Immunol. 15, 1512353. 10.3389/fimmu.2024.1512353 39867908 PMC11757137

[B211] Suraweera A. O’Byrne K. J. Richard J. J. (2025). Epigenetic drugs in cancer therapy. Cancer Metastasis. Rev. 44, 37. 10.1007/s10555-025-10253-7 40011240 PMC11865116

[B175] Swiecki M. Colonna M. (2011). Type I interferons: diversity of sources, production pathways and effects on immune responses. Curr. Opin. Virol. 1, 463–475. 10.1016/j.coviro.2011.10.026 22440910 PMC3572907

[B176] Szabo Q. Bantignies F. Cavalli G. (2019). Principles of genome folding into topologically associating domains. Sci. Adv. 5, eaaw1668. 10.1126/sciadv.aaw1668 30989119 PMC6457944

[B177] Tachon G. Chong-Si-Tsaon A. Lecomte T. Junca A. Frouin É. Miquelestorena-Standley E. (2022). HSP110 as a diagnostic but not a prognostic biomarker in colorectal cancer with microsatellite instability. Front. Genet. 12, 769281. 10.3389/fgene.2021.769281 35047001 PMC8762103

[B178] Tajudeen Y. John E. Hewage A. S. Liman U. U. Caleb O. (2025). Epigenetic modifications in cancer etiology, diagnosis and therapy. Asian J. Biol. Sci. 18, 516–532. 10.3923/ajbs.2025.516.532

[B179] Taylor K. Loo Yau H. Chakravarthy A. Wang B. Shen S. Y. Ettayebi I. (2020). An open-label, phase II multicohort study of an oral hypomethylating agent CC-486 and durvalumab in advanced solid tumors. J. Immunotherapy Cancer 8, e000883. 10.1136/jitc-2020-000883 32753546 PMC7406114

[B180] Tereda A. Fatima F. Javaid H. Mehmood Q. Shahid F. Saddique M. N. (2025). Combination of nivolumab plus ipilimumab in microsatellite instability-high metastatic colorectal cancer: a systematic review and meta-analysis. J. Clin. Oncol. 43, e15516. 10.1200/JCO.2025.43.16_suppl.e15516 40700037

[B181] Topper M. J. Vaz M. Marrone K. A. Brahmer J. R. Baylin S. B. (2020). The emerging role of epigenetic therapeutics in immuno-oncology. Nat. Rev. Clin. Oncol. 17, 75–90. 10.1038/s41571-019-0266-5 31548600 PMC7254932

[B182] UCSC Genome Browser (2025). Available online at: https://genome.ucsc.edu/(Accessed August 18, 2025).

[B183] Vedeld H. M. Goel A. Lind G. E. (2018). Epigenetic biomarkers in gastrointestinal cancers: the current state and clinical perspectives. Semin. Cancer Biol. 51, 36–49. 10.1016/j.semcancer.2017.12.004 29253542 PMC7286571

[B184] Wang Z. Dong C. (2019). Gluconeogenesis in cancer: function and regulation of PEPCK, FBPase, and G6Pase. Trends Cancer 5, 30–45. 10.1016/j.trecan.2018.11.003 30616754

[B185] Wang L. Lankhorst L. Bernards R. (2022). Exploiting senescence for the treatment of cancer. Nat. Rev. Cancer 22, 340–355. 10.1038/s41568-022-00450-9 35241831

[B186] Wang T. Liu W. Shen Q. Tao R. Li C. Shen Q. (2023). Combination of PARP inhibitor and CDK4/6 inhibitor modulates cGAS/STING‐dependent therapy‐induced senescence and provides “one‐two punch” opportunity with anti‐PD‐L1 therapy in colorectal cancer. Cancer Sci. 114, 4184–4201. 10.1111/cas.15961 37702298 PMC10637067

[B187] Wang D. Chen K. Wang Z. Wu H. Li Y. (2024a). Research progress on interferon and cellular senescence. FASEB J. 38, e70000. 10.1096/fj.202400808RR 39157951

[B188] Wang Y. Wang C. Zhong R. Wang L. Sun L. (2024b). Research progress of DNA methylation in colorectal cancer. Mol. Med. Rep. 30, 1–12. 10.3892/mmr.2024.13278 PMC11240861 38963030

[B189] Weisenberger D. J. Siegmund K. D. Campan M. Young J. Long T. I. Faasse M. A. (2006). CpG island methylator phenotype underlies sporadic microsatellite instability and is tightly associated with BRAF mutation in colorectal cancer. Nat. Genet. 38, 787–793. 10.1038/ng1834 16804544

[B190] Weren R. D. Venkatachalam R. Cazier J. Farin H. F. Kets C. M. de Voer R. M. (2015). Germline deletions in the tumour suppressor gene FOCAD are associated with polyposis and colorectal cancer development. J. Pathol. 236, 155–164. 10.1002/path.4520 25712196 PMC6681464

[B191] Witcher M. Emerson B. M. (2009). Epigenetic silencing of the p16INK4a tumor suppressor is associated with loss of CTCF binding and a chromatin boundary. Mol. Cell 34, 271–284. 10.1016/j.molcel.2009.04.001 19450526 PMC2723750

[B192] Wyld L. Bellantuono I. Tchkonia T. Morgan J. Turner O. Foss F. (2020). Senescence and cancer: a review of clinical implications of senescence and senotherapies. Cancers 12, 2134. 10.3390/cancers12082134 32752135 PMC7464619

[B193] Xing X. Cai W. Shi H. Wang Y. Li M. Jiao J. (2013). The prognostic value of CDKN2A hypermethylation in colorectal cancer: a meta-analysis. Br. J. Cancer 108, 2542–2548. 10.1038/bjc.2013.251 23703248 PMC3694241

[B194] Xu X.-L. Yu J. Zhang H.-Y. Sun M.-H. Gu J. Du X. (2004). Methylation profile of the promoter CpG islands of 31 genes that may contribute to colorectal carcinogenesis. World J. Gastroenterol. 10, 3441–3454. 10.3748/wjg.v10.i23.3441 15526363 PMC4576225

[B195] Xu Y. Liu K. Li C. Li M. Zhou X. Sun M. (2024). Microsatellite instability in mismatch repair proficient colorectal cancer: clinical features and underlying molecular mechanisms. eBioMedicine 103, 105142. 10.1016/j.ebiom.2024.105142 38691939 PMC11070601

[B196] Yang X. R. Rotunno M. Xiao Y. Ingvar C. Helgadottir H. Pastorino L. (2016). Multiple rare variants in high-risk pancreatic cancer related genes may increase risk for pancreatic cancer in a subset of patients with and without germline CDKN2A mutations. Hum. Genet. 135, 1241–1249. 10.1007/s00439-016-1715-1 27449771 PMC5152573

[B197] Yang L. Ma D. Cao Y. Li D. Zhou X. Feng J. (2021). PRMT5 functionally associates with EZH2 to promote colorectal cancer progression through epigenetically repressing CDKN2B expression. Theranostics 11, 3742–3759. 10.7150/thno.53023 33664859 PMC7914347

[B198] Yang L. Chen X. Lee C. Shi J. Lawrence E. B. Zhang L. (2023). Functional characterization of age-dependent p16 epimutation reveals biological drivers and therapeutic targets for colorectal cancer. J. Exp. Clin. Cancer Res. 42, 113. 10.1186/s13046-023-02689-y 37143122 PMC10157929

[B199] Yap K. L. Li S. Muñoz-Cabello A. M. Raguz S. Zeng L. Mujtaba S. (2010). Molecular interplay of the non-coding RNA ANRIL and methylated histone H3 Lysine 27 by polycomb CBX7 in transcriptional silencing of INK4a. Mol. Cell 38, 662–674. 10.1016/j.molcel.2010.03.021 20541999 PMC2886305

[B200] Yu Q. Katlinskaya Y. V. Carbone C. J. Zhao B. Katlinski K. V. Zheng H. (2015). DNA damage-induced type I interferon promotes senescence and inhibits stem cell function. Cell Rep. 11, 785–797. 10.1016/j.celrep.2015.03.069 25921537 PMC4426031

[B201] Zhang T. (2025). An open-label, single-arm, exploratory Study of sintilimab in combination with Bevacizumab and decitabine for the treatment of advanced pMMR/MSS colorectal cancer in third-line or later settings. clinicaltrials.gov. Available online at: https://clinicaltrials.gov/study/NCT07007767 (Accessed August 19, 2025).

[B202] Zhang Y. Hyle J. Wright S. Shao Y. Zhao X. Zhang H. (2019). A cis-element within the ARF locus mediates repression of p16INK4A expression *via* long-range chromatin interactions. Proc. Natl. Acad. Sci. U. S. A. 116, 26644–26652. 10.1073/pnas.1909720116 31818950 PMC6936709

[B203] Zhao R. Choi B. Y. Lee M.-H. Bode A. M. Dong Z. (2016). Implications of genetic and epigenetic alterations of CDKN2A (p16(INK4a)) in cancer. EBioMedicine 8, 30–39. 10.1016/j.ebiom.2016.04.017 27428416 PMC4919535

[B204] Zhao G. Li H. Yang Z. Wang Z. Xu M. Xiong S. (2019). Multiplex methylated DNA testing in plasma with high sensitivity and specificity for colorectal cancer screening. Cancer Med. 8, 5619–5628. 10.1002/cam4.2475 31407497 PMC6745865

[B205] Zhong Y. Lu K. Zhu S. Li W. Sun S. (2018). Characterization of methylthioadenosin phosphorylase (MTAP) expression in colorectal cancer. Artif. Cells Nanomed Biotechnol. 46, 2082–2087. 10.1080/21691401.2017.1408122 29268653

[B206] Zhou L. Zhang Y. Wang Y. Zhang M. Sun W. Dai T. (2020). A dual role of type I interferons in Antitumor immunity. Adv. Biosyst. 4, e1900237. 10.1002/adbi.201900237 33245214

[B207] Zhou Y. Nakajima R. Shirasawa M. Fikriyanti M. Zhao L. Iwanaga R. (2023). Expanding roles of the E2F-RB-p53 pathway in tumor suppression. Biol. (Basel) 12, 1511. 10.3390/biology12121511 38132337 PMC10740672

